# Pan-Cancer Pyroptosis Analyses Identified Novel Immunology and Chemotherapy-Related Prognostic Signatures in Cancer Subtypes

**DOI:** 10.1155/2022/6609297

**Published:** 2022-06-20

**Authors:** Canrong Li, Cha Lin, Xiaoduo Xie

**Affiliations:** ^1^Department of Biochemistry, School of Medicine, Shenzhen Campus of Sun Yat-Sen University, Guangzhou, China; ^2^Neurobiology Research Center, School of Medicine, Shenzhen Campus of Sun Yat-Sen University, Guangzhou, China; ^3^State Key Laboratory of Pharmaceutical Biotechnology, Nanjing University, Nanjing, China

## Abstract

Despite mounting evidence linking pyroptotic cell death to tumor growth, the clinical significance and disease mechanism of pyroptosis in cancer remain uncertain. In this study, we established a unique gene signature (*π* signature) that can be used as a predictive and prognostic tool in pyroptosis-related cancer subtypes. We found that the 13 core pyroptosis genes exerted opposite prognostic effects in different cancer types, which were subgrouped as pyroptosis positively related cancer and pyroptosis negatively related cancer. Subsequently, *π* signature was identified separately from the hub genes in pyroptosis positively related cancer and pyroptosis negatively related cancer subtypes. It was shown that *π* signature was well correlated with patient survival, pathological stages, tumor lymphocyte infiltration, and immunotherapy response. *π* signature was also applied as a predictive tool for chemotherapy drug responses and used as an independent factor for patient overall survival prediction. In short, this elaborated genetic signature could help us understand the oncogenic mechanism and pave the way for further therapeutic strategies based on pyroptosis.

## 1. Introduction

Programmed cell death (PCD), such as apoptosis, pyroptosis, and autophagic cell death, is regulated by specific signaling pathways that mediate the process of cell mortality induced by intrinsic or extrinsic factors. Many oncogenic or tumor suppressor molecules in these pathways have been identified and are being applied as drug targets in cancer therapeutics [1, 2]. Pyroptosis is morphologically characterized by bubble-like cell swelling and plasma membrane rupture. When compared with nonlytic apoptotic or autophagy-related cell death, pyroptosis belongs to immunogenic cell death (ICD) associated with the release of proinflammatory cytokines and priming of immune responses [3]. Recent advances have revealed that pyroptosis is directly executed by gasdermin (GSDM) family proteins including gasdermin A (GSDMA), gasdermin B (GSDMB), gasdermin C (GSDMC), gasdermin D (GSDMD), gasdermin E (GSDME/DFNA5), and pejvakin (PJVK/DFNB59) [4]. These paralogues share strong sequence similarity in their cytotoxic N-terminal domain, which is repressed by intramolecular C-terminal domain. A flexible linker between the N- and C-terminal domains is proteolytically activated by upstream proteases, mostly by caspases [5, 6]. The canonic pyroptosis pathway, originally discovered in macrophages, is mediated by caspase-1 (CASP1), which undergoes self-cleavage activation after assembling into inflammasome (CASP1/ASC/Pyrin/AIM2/NAIP/NLRC4/NLRP complex) in response to pathogen-associated molecular patterns (PAMPs) [7]. The activated CASP1 then cleaves GSDMD, pro-IL-18, and IL-1*β*, and the cleaved GSDMD could eventually trigger pyroptosis [8, 9]. In the noncanonical pyroptosis pathway, lipopolysaccharide (LPS) from Gram-negative bacteria directly binds to and activates CASP4/5 or mouse CASP11, which also cleaves GSDMD to induce pyroptosis [10, 11]. In response to certain stimuli, other proteases, such as CASP3, CASP8, Granzyme A (GZMA), and Granzyme B (GZMB) [12–15], can also activate GSDMs, resulting in pyroptosis.

Pyroptosis is a ubiquitous biological process involved in many different cell types responding to different PAMPs or DAMPs [16, 17]. Extensive evidence implies that it plays an important role in many cancers. Pyroptotic factors such as CASPs or GSDMs activation or proinflammatory cytokine-related immune responses are significantly associated with tumorigenesis and cancer progression [3, 18]. Apoptotic and pyroptotic cell death suppress tumor progression, which means pyroptosis could be a feasible way to inhibit tumor growth. Indeed, various tumoricidal substances, such as chemotherapy drugs or granzymes released from NK cells or cytotoxic T cells, could eliminate tumors via canonical or noncanonical pyroptotic pathways [15, 19]. However, it has also been reported that pyroptosis promotes the progression of some cancer types, which is attributed to its proinflammatory effects. As a type of ICD, pyroptosis happening in tumor or nontumor cells may induce a tumor's favorable microenvironment to promote disease progression [20, 21]. Importantly, pyroptosis-induced immune responses are a double-edged sword since they could also help to activate or normalize patients' compromised immune systems and modulate the tumor microenvironment (TME) through DAMP-induced tumor infiltrating lymphocytes (TILs) [14, 15, 18]. Immunotherapy such as immune checkpoint inhibition (ICI) has achieved remarkable success in the clinics [22–24]. Studies have shown that the efficacy of ICI can be substantially improved when combined with irradiation-induced or chemotherapy-induced tumor pyroptotic cell death [15, 18, 25, 26]. Thus, it would be interesting to test if pyroptosis inducible compounds could be combined with ICI immunotherapy to improve efficacy. Such combinational therapies are therapeutically necessary, especially for patients with immunologically “cold” tumors.

There is no conclusive evidence that pyroptosis induction improves cancer prognosis, and the precise roles of pyroptosis and the genetic background required for antitumor immunity remain unknown. To conquer this ambiguity, we analyzed several public tumor databases and developed integrated prediction models for different pyroptosis-related cancer subtypes with elaborated gene signatures.

## 2. Results

### 2.1. Pyroptosis Core Genes Are Strongly Correlated with Tumor Immunity

**Figure 1:**
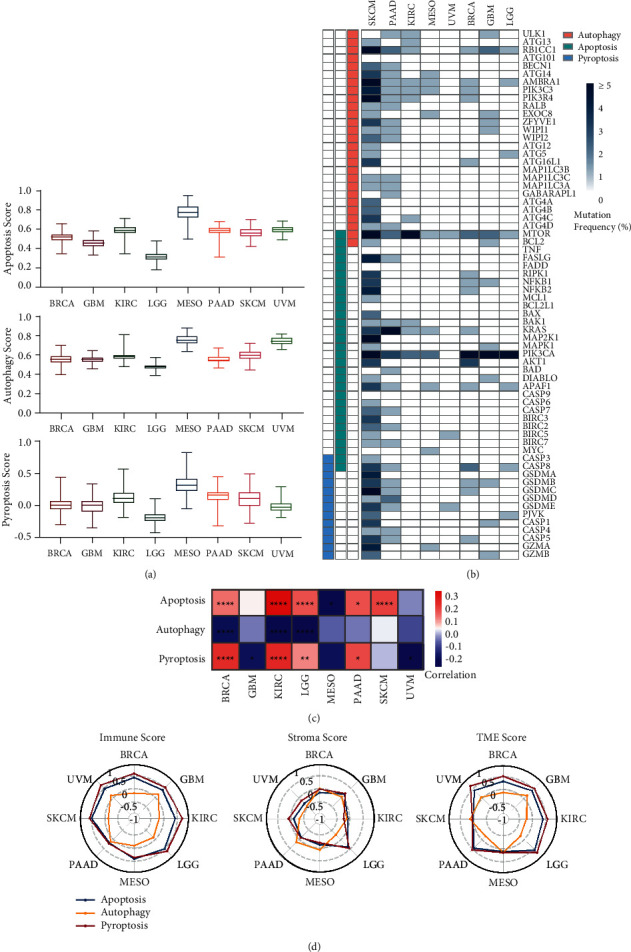
Thirteen pyroptosis core genes strongly correlated with tumor immunity. (a) Box plots showed the 3 programmed cell death (PCD) scores (apoptosis, autophagy, and pyroptosis scores) of cases in each cancer type. (b) The heatmap showed the mutation frequency of genes related to apoptosis, autophagy, and pyroptosis. (c) The heatmap showed the correlation between the tumor mutational burden (TMB) and 3 PCD scores in each cancer type. ^*∗*^*P* < 0.05, ^*∗∗*^*P* < 0.01, ^*∗∗∗∗*^*P* < 0.0001. (d) Radar charts showed the correlation between 3 PCD scores and 3 tumor immunity-related scores calculated by XCELL (Immune score, Stroma score, and tumor microenvironment (TME) score), and the number denotes Pearson correlation coefficients (*R*).

To evaluate the predictive or prognostic values of pyroptosis-related molecules in cancer, we defined 13 core genes that are directly involved in pyroptosis signaling (Supplementary Figure 1A). The mRNA expression, somatic mutation, and the follow-up information were extracted from 8 different cancer types in The Cancer Genome Atlas (TCGA) database (Table 1). For comparison, we selected the other 2 groups of PCD core genes (Supplementary Figures 1A-B). PCD scores were calculated for each patient from 8 cancer types, including breast invasive carcinoma (BRCA), glioblastoma multiforme (GBM), kidney renal clear cell carcinoma (KIRC), lower grade glioma (LGG), skin cutaneous melanoma (SKCM), mesothelioma (MESO), pancreatic adenocarcinoma (PAAD), and uveal melanoma (UVM) (Figure 1(a), Supplementary Figure 1C). As shown in Supplementary Figure 2, higher expression of 13 pyroptosis core genes resulted in the higher pyroptosis score. No difference was observed for tumor somatic mutation frequencies among the 3 groups of core genes, although a higher mutation frequency existed in some cancer types such as SKCM and PAAD for almost all analyzed genes (Figure 1(b)). Notably, tumor mutational burden (TMB) significantly correlated with pyroptosis and apoptosis scores of patients in 7 out of 8 cancer types (Figure 1(c)). As expected, the pyroptosis score, compared with the other 2 PCD scores, had the strongest correlation with both the immune score and the TME score (Figure 1(d), Supplementary Figures 1D-F). These results indicated that the 13 pyroptosis core genes were closely correlated with tumor immunity, consistent with the immunogenic nature of pyroptosis.

### 2.2. Classification of Cancer Subtypes by the Pyroptosis Score

**Figure 2:**
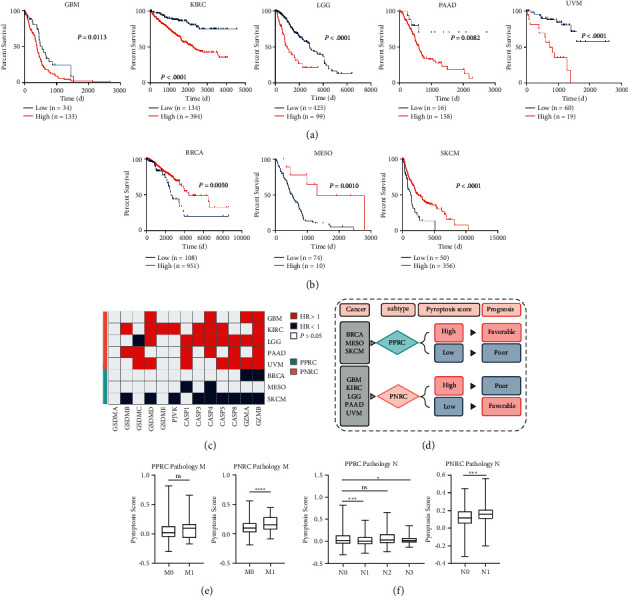
The classification of cancer subtypes by the opposite correlation between the pyroptosis score and prognosis. (a-b) Kaplan–Meier (KM) survival curves for pyroptosis positively related cancer (PPRC) (a) and pyroptosis negatively related cancer (PNRC) (b). The high (red) and low (blue) pyroptosis scores were stratified by the optimal cutoff values calculated by the R package “survminer.” (c) The heatmap showed the hazard ratio (HR) of each pyroptosis core gene in 8 cancer types (*P* > 0.05 was excluded). Eight cancer types fell into two subgroups based on the significant difference in the prognostic value of pyroptosis core genes. (d) A schematic diagram of the PPRC and PNRC subtype definitions. (e, f) Association between the pyroptosis score and tumor pathological stages featured by pathology M (e) or pathology N (f). Kruskal–Wallis one-way ANOVA was used to calculate the global *P* value among 4 stages of PPRC pathology N, and Student's *t*-test was used for pairwise comparisons. ^*∗*^*P* < 0.05, ^*∗∗∗*^*P* < 0.001, ^*∗∗∗∗*^*P* < 0.0001; ns, not significant; M0, cancer has not spread to other parts of the body; M1, cancer has spread to other parts of the body; N0, no evidence of cancer in the regional lymph nodes; N1/N2/N3, number and location of lymph nodes that contain cancer, higher the number, the more lymph nodes that contain cancer.

Next, we grouped patients with high and low pyroptosis scores (Table S1), and Kaplan–Meier (KM) curves revealed that patients from GBM, KIRC, LGG, PAAD, and UVM with high pyroptosis scores showed poor outcomes (Figure 2(a)), while patients from BRCA, MESO, and SKCM with high pyroptosis scores had favorable outcomes (Figure 2(b)). Each of the 13 core pyroptosis genes was further assessed for their prognostic effects in different cancer patients, and consequently, the pyroptosis genes' prognostic distinctions divided cancers into 2 subgroups: the BRCA, MESO, and SKCM subgroup with a hazard ratio (HR) > 1 for most of the 13 pyroptosis core genes, and the GBM, KIRC, LGG, PAAD, and UVM subgroup with an HR < 1 for most of the 13 pyroptosis core genes (Figure 2(c)). As such, we subgrouped 8 cancer types into two following subtypes: pyroptosis positively related cancer (PPRC) and pyroptosis negatively related cancer (PNRC) (Figure 2(d)). Association between the pyroptosis score and tumor clinical features further confirmed this notion. PNRC patients with a higher pyroptosis score showed more advanced pathology in both the M and N categories (Figures 2(e) and 2(f)), whereas most PPRC patients with a high pyroptosis score showed no or less progressive pathology (Figures 2(e) and 2(f)). Overall, two cancer subtypes (PPRC and PNRC) were identified with the opposite correlation between pyroptosis and prognosis.

### 2.3. Identification of *π* Signature in PPRC and PNRC Subtypes

**Figure 3:**
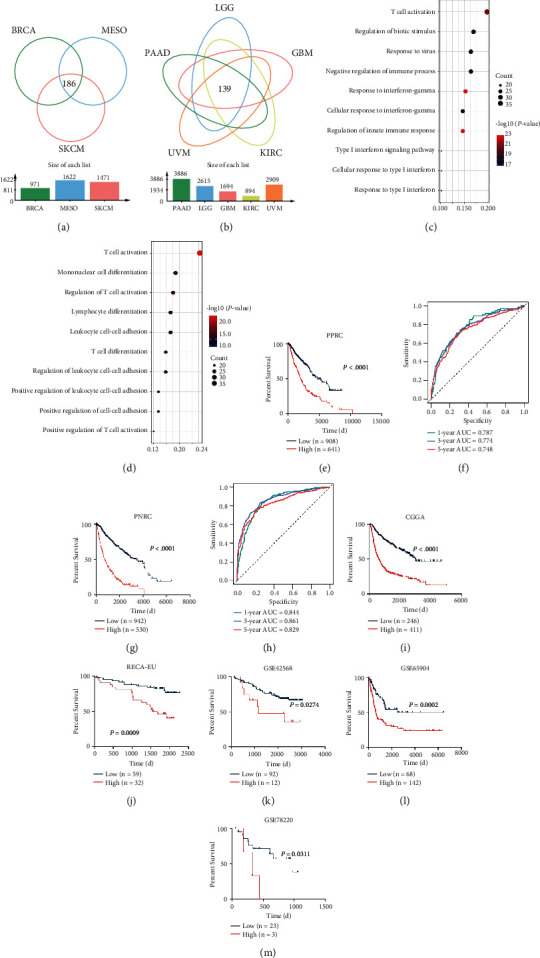
Construction of *π* signature in PPRC and PNRC subtypes. (a) The Venn diagram showed the 186 intersected differential expressed genes (iDEGs) from PPRC samples. (b) The Venn diagram showed the 139 iDEGs from PNRC samples. (c, d) Top 10 enriched gene ontology (GO) pathways in the iDEGs from PPRCs (c) and PNRCs (d). The *x*-axis indicated the gene ratio within each GO term. (e) The KM survival curve based on the *π* score from PPRC in TCGA cohort. The survival curve was compared using the log-rank test. High (red) and low (blue) PP scores were determined by the R package “survminer.” (f) 1-, 3-, and 5-year overall survival (OS) receiver operating characteristic (ROC) curves in the PPRC demonstrated relatively satisfactory predictive performance. The area under the curve (AUC) values were 0.787 (95% CI: 73.24–84.15) at 1 year, 0.774 (95% CI: 73.71–81.18) at 3 years, and 0.748 (95% CI: 70.88–78.75) at 5 years. (g) The KM survival curve based on the *π* score from PNRC in TCGA cohort. The survival curve was compared using the log-rank test. High (red) and low (blue) PN scores were determined by the R package “survminer.” (h) The 1-, 3-, and 5-year overall survival ROC curves in PNRC demonstrated reasonably good predictive performance. AUC values of ROC curves were 0.844 (95% CI: 81.47–87.27) at 1 year, 0.861 (95% CI: 83.66–88.63) at 3 years, and 0.829 (95% CI: 79.76–86.13) at 5 years. (i–m) *π* signature was validated with five cancer cohorts. KM survival curves showed the OS of patients of high (red) and low (blue) *π* scores in CGGA (i), RECA-EU (j), GSE42568 (k), GSE65904 (l), and GSE78220 (m) cohorts.

Hub genes of PPRC and PNRC were identified by intersecting differential expressed genes (DEGs) (Supplementary Figure 3) from the PPRC subgroup or PNRC subgroup separately (Figures 3(a) and 3(b)), and as a result, 186 hub genes of PPRC and 139 of PNRC were identified (Supplementary Figures 4A-B, Table S2) and showed strong correlations with the pyroptosis score (Supplementary Figures 4C-D). Gene ontology (GO) enrichment analysis of hub genes enriched 308 biological processes (BPs) in PPRC and 207 BPs in PNRC, respectively (Tables S3-4), and the top ten enriched BPs from both subtypes were related to immune response, with T cell activation at the top (Figures 3(c) and 3(d)). BPs enriched in PPRC were mainly from interferon signaling (Figure 3(c)), while BPs enriched in PNRC were mainly from lymphocyte cell-cell adhesion or differentiation signaling (Figure 3(d)). Univariate Cox regression analysis revealed that 132 of 186 hub genes from PPRC and 85 of 139 hub genes from PNRC were significantly related to the overall survival (OS) of patients (*P* < 0.05) (Tables S5-6). We performed Lasso Cox regression analysis and constructed an 11-gene signature for PPRC and another 39-gene signature for PNRC (Supplementary Figures 5A-D, Tables S7-8). Thereafter, these signatures were designated as “*π*” signature (onset syllable of pyroptosis) and quantified as *π* score (PP score for PPRC and PN score for PNRC). High and low *π* score patients were grouped (Table S9), and KM analyses revealed that the high PP score group had poor prognosis (*P* < 0.0001) in PPRC patients (Figure 3(e), Supplementary Figures 6A-C). Receiver operating characteristic (ROC) curves showed the accuracy of the PP score (Figure 3(f)). The area under the curve (AUC) values for the OS of PPRC patients were 0.787 at 1 year, 0.774 at 3 years, and 0.748 at 5 years (Figure 3(f)). Similar results were obtained from PNRC patients (Figure 3(g), Supplementary Figures 6D-H), and AUC values were 0.844 at 1 year, 0.861 at 3 years, and 0.829 at 5 years (Figure 3(h)). We further confirmed the predictive ability of *π* score in 5 validated cohorts (Table 1). By comparing the AUC of *π* score to previously established pyroptosis signatures, we found the AUC of our *π* signature was higher than most of them (Table 2). These results demonstrate that *π* signature is a strong prognostic marker for tumors with different genetic backgrounds.

### 2.4. The Prognostic and Predictive Relevance of *π* Signature

**Figure 4:**
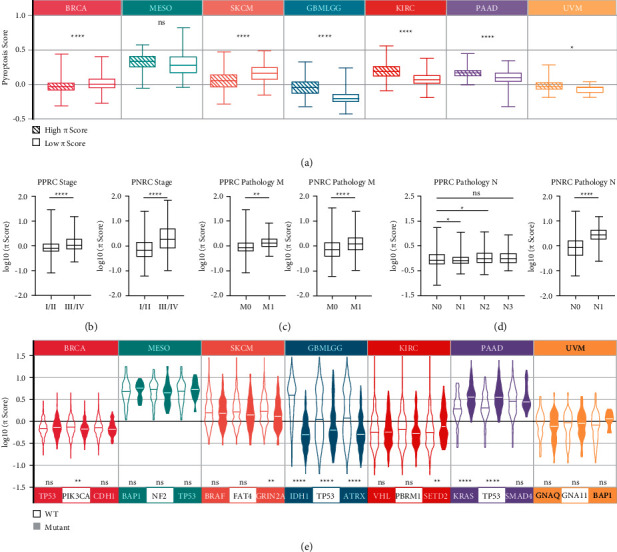
Prognostic and predictive relevance analyses of *π* signature. (a) The relationship between the pyroptosis score and the *π* score in different cancer types. ^*∗*^*P* < 0.05, ^*∗∗∗∗*^*P* < 0.0001; ns, not significant.(B-D) Association of *π* score with tumor pathological stages featured by tumor stages (b), pathology M stages (c), pathology N stages (d), Kruskal–Wallis one-way ANOVA was used to calculate the global *P* value among 4 stages of PPRC pathology N, and Student's *t*-test was used for pairwise comparisons. ^*∗∗*^*P* < 0.01, ^*∗∗∗∗*^*P* < 0.0001; ns, not significant; I/II, stage I and stage II; III/IV, stage III and stage IV; M0, cancer has not spread to other parts of the body; M1, cancer has spread to other parts of the body; N0, no evidence of cancer in the regional lymph nodes; N1/N2/N3, number and location of lymph nodes that contain cancer, higher the number, the more lymph nodes that contain cancer. (e) Association of *π* score with the top 3 mutated genes in specific cancer types. ^*∗*^*P* < 0.05, ^*∗∗*^*P* < 0.01, ^*∗∗∗*^*P* < 0.001, ^*∗∗∗∗*^*P* < 0.0001; ns, not significant; WT, wild-type.

Correlation analysis showed that the PP score was negatively correlated with pyroptosis genes, whereas the PN score was positively correlated with the pyroptosis genes (Supplementary Figure 7). This is expected as the PP score behaved oppositely to the pyroptosis score in the prognosis of PPRC patients (Figures 2(b) and 3(e)), while the PN score behaved consistently to the pyroptosis score in the prognosis of PNRC patients (Figures 2(c) and 3(g)). Indeed, high pyroptosis scores in BRCA and SKCM were associated with a low PP score, while high pyroptosis scores in all PNRC patients were associated with a high PN score (Figure 4(a)). Although MESO patients did not show statistically significant correlations like those of BRCA and SKCM, most of the pyroptosis core genes did show negative correlations with the PP score (Figure 4(a), Supplementary Figure 7). Consistent with Figure 3, these results consolidated the prognostic value of *π* signature. Besides, *π* score in PPRC and PNRC were significantly higher in more advanced pathological stages (Figures 4(b)–4(d)), implicating remarkable predictive relevance of *π* signature. Oncogenic mutations profoundly affect tumorigenesis. However, the prognosis of these mutations in different cancer types is mostly unclear or controversial. To clarify, we selected the top 3 mutated genes of each cancer (Table S10), and significant changes of *π* score were observed upon the mutation happened, such as isocitrate dehydrogenase 1 (IDH1), tumor protein P53 (TP53), or ATRX chromatin remodeler (ATRX) mutation in GBMLGG exhibited lower PN score, SET domain containing 2 (SETD2) mutation in KIRC and Kirsten rat sarcoma oncogene homolog (KRAS) or TP53 mutation in PAAD-exhibited higher PN scores, and phosphatidylinositol-4,5-bisphosphate 3-kinase catalytic subunit alpha (PIK3CA) or glutamate ionotropic receptor NMDA type subunit 2A (GRIN2A) mutation in PPRC patients exhibited higher PP scores (Figure 4(e)). Importantly, mutation data were consistent with the survival analyses as shown in Supplementary Figure 8. These results agree with the current molecular prognosis paradigm that some mutations of oncogenes or tumor suppressor genes are not necessarily prognostic detrimental (more details in the Discussion section). Thus, our findings suggested that *π* signature could be applied as a prognostic and predictive reference for different cancer subtypes.

### 2.5. *π* Signature Is Related to Tumor Immunity and Immunotherapy Response

**Figure 5:**
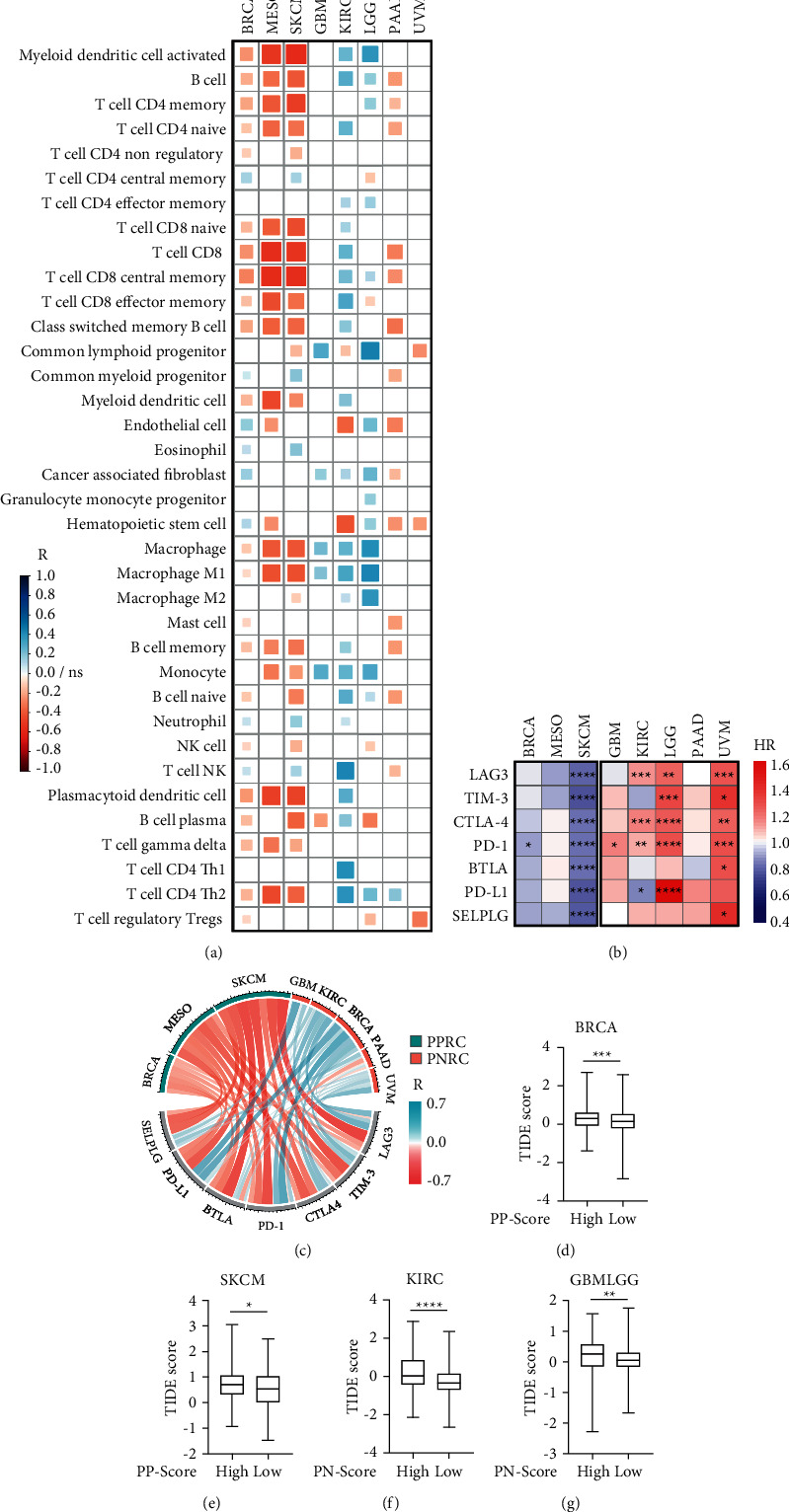
The relationship between the *π* signature and the tumor immunity. (a) Pearson corrplot showed the correlation between immune cell infiltration and patients' *π* score. *R*, Pearson correlation coefficients; ns, not significant. (b) The heatmap showed different prognostic effects of the checkpoint molecules' expression in PPRC and PNRC subtypes. HR, hazard ratio; ^*∗*^*P* < 0.05, ^*∗∗*^*P* < 0.01, ^*∗∗∗*^*P* < 0.001, ^*∗∗∗∗*^*P* < 0.0001. (c) The chord diagram showed the positive (roseate) or negative (cyan) correlation between the expression of immune checkpoint molecules and the *π* score. *R*, Pearson correlation coefficients. (d, e) Association of the Tumor Immune Dysfunction and Exclusion (TIDE) score with the PP score in BRCA (d) and SKCM (e) subgroups of patients. ^*∗*^*P* < 0.05, ^*∗∗∗*^*P* < 0.001. (f, g) Association of the TIDE score with the PN score in KIRC (f) and glioma (both GBM and LGG) (g) subgroups of patients. ^*∗∗*^*P* < 0.01, ^*∗∗∗∗*^*P* < 0.0001.

To pinpoint the mechanisms of how pyroptosis contributes to tumor immunity (Figure 1(d)), we analyzed the TILs in patients. 36 types of TILs were fractionated by XCELL, and we found that *π* score had a significant association with general immune cell infiltration (Figure 5(a)). Specifically, the PP score was negatively correlated with the infiltration of dendritic cell (DC), B cell, CD4 T cell, CD8 T cell, and macrophage in PPRC patients, whereas the PN score was positively correlated with several TILs in GBM, KIRC, and LGG but negatively correlated with TILs in PAAD and UVM (Figure 5(a)). In short, *π* signature differentially reflected TME in cancer subtypes. As shown in Table S2, programmed cell death 1 (PD-1) and CD274 molecule (PD-L1) were PPRC hub genes, and cytotoxic T-lymphocyte associated protein 4 (CTLA-4) was a PNRC hub gene. For further analyses, we selected well-known immune checkpoint molecules: PD-1, PD-L1, lymphocyte activating 3 (LAG3), hepatitis A virus cellular receptor 2 (TIM-3), CTLA-4, B and T lymphocyte associated (BTLA), and selectin P ligand (SELPLG), and we found they exhibited opposite prognostic effects in PPRC and PNRC subtypes (Figure 5(b)), implying possible linkage between pyroptosis and checkpoint molecules in different cancer subtypes. Consistently, the expression of checkpoint molecules was negatively correlated with the PP score and positively correlated with the PN score, showing the opposite mechanistic association of checkpoint molecules with *π* signature (Figure 5(c)). Subsequent analysis correlated the immunotherapy response with *π* signature, as we observed that a higher *π* score in patients showed a higher TIDE score (poor immunotherapy response) (Figures 5(d)–5(g)), suggesting that *π* signature could be an auxiliary predictive tool for immunotherapy response.

### 2.6. *π* Signature Is a Predictive Tool for Chemotherapy Drug Responses

**Figure 6:**
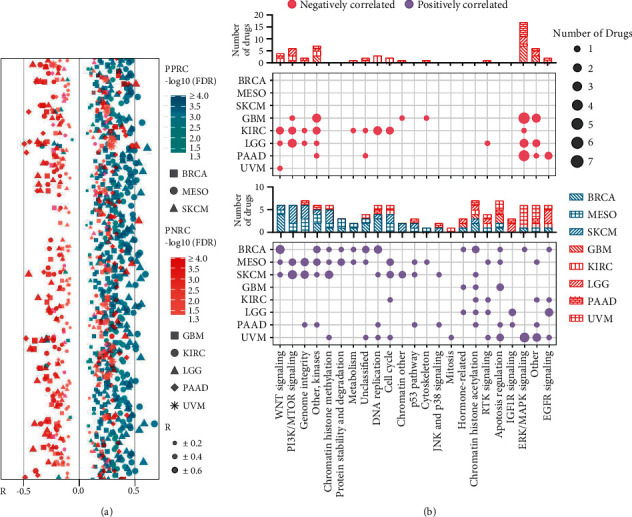
The *π* signature function as a predictive tool for chemotherapy drug responses. (a) Pearson analysis of the correlation between IC50 of 198 chemotherapy drugs and the *π* score, the PP score (cyan), or PN score (roseate). FDR, false discovery rate; *R*, Pearson correlation coefficients. (b) Bubble plots showed the signaling pathways targeted by the top 20 correlated drugs. The sensitivity of these drugs is related to *π* score in each cancer. The size of the point indicated the number of drugs.

Chemotherapy drugs such as small molecule inhibitors are still the mainstream in cancer treatment. Pyroptosis induced by chemotherapy drugs could elicit immune responses or TME modulation and affect the efficacy of immunotherapy when combined with immune checkpoint inhibitors. Hence, the association between *π* scores and IC50 of 198 drugs was assessed, and we found the PP score was positively correlated with IC50 of most drugs (177, 161, and 170 of 198 drugs showed positive correlation in BRCA, MESO, and SKCM, respectively, but only 4, 2, and 10 of 198 drugs showed negative correlation) (Figure 6(a)), indicating lower therapeutic efficacy of most drugs in higher PP score patients. In contrast, 26, 34, 54, 95, and 17 of 198 drugs showed positive correlation in GBM, KIRC, LGG, PAAD, and UVM, respectively, while 56, 94, 81, 36, and 1 of 198 drugs showed negative correlation (Figure 6(a), Table S11).

Next, signaling pathways targeted by the top 20 correlated drugs were analyzed in each cancer, and we found that high PN score patients were more sensitive to drugs targeting the WNT signaling, ERK/MAPK signaling, and PI3K/MTOR signaling pathway (Figure 6(b) A). In contrast, high PP score patients were more resistant to drugs targeting the WNT signaling, chromatin histone methylation signaling, protein stability, and degradation signaling pathway, while high PN score patients were more resistant to drugs targeting the EGFR signaling and chromatin histone acetylation signaling pathway (Figure 6(b) B). As such, we tried to predict the chemotherapy drug responses using *π* score in different cancer subtypes. As we did in Table 3, the top 5 positively or negatively correlated drugs for each cancer type were listed. Those drugs in the left column could be potentially applied to patients with a low *π* score (stable patients), and those drugs in the right column could be potentially applied to patients with a high *π* score (progressive patients) (Table 3). In summary, *π* signature could be a predictive tool for chemotherapy drug responses in different cancer subtypes.

### 2.7. Application of *π* Signature as an Independent Predictive Factor for OS Prediction

To further evaluate if *π* signature could be used as an independent prognostic parameter for OS prediction, univariate Cox regression analysis was conducted, and the PP score or PN score, as well as other clinicopathologic parameters such as age and tumor stage, were independent prognostic factors affecting the OS of cancer patients in both PPRC and PNRC (Supplementary Figures 9A-B). We then developed 2 nomogram models for PPRC and PNRC patients, respectively (Supplementary Figures 10A-B). In these models, the score of each parameter was identified by plotting a straight line upwards to cross the point axis and the total points of each patient as the sum of all individual parameter scores. The survival rate of patients in 1, 3, and 5 years was estimated by plotting a perpendicular line downwards from the total point axis to the resulting axis. The concordance index (C-index) was 0.800 for the PPRC nomogram model and 0.803 for PNRC. Favorable calibrations were confirmed in Supplementary Figure 9C-H, suggesting satisfactory consistency between nomogram model predictions and practical observations in 1-, 3-, and 5-year OS of patients. In conclusion, *π* signature is an independent prognostic factor in the integrated nomogram models providing satisfactory prediction of a patient's OS in cancer subtypes.

## 3. Discussion

Increasing evidence has implicated the essential and versatile roles of pyroptosis in various cancers with different genetic backgrounds, and it is commonly acknowledged that pyroptosis functions as a double-edged sword in cancer patients due to its divergent effects in tumor cell homeostasis, tumor immunity, and TME modulation. Thus, to determine the precise involvement of pyroptosis in individual cancers, a systematic study of pyroptosis in pan-cancer is imperative. Furthermore, because tumor cells respond differently to various pyroptosis-based treatments, identifying the cancer type-specific gene signature as a precise tool for predicting disease progression and patient prognosis is vital.

To address these issues, we first investigated whether pyroptosis activity, as manifested by the expression of 13 core genes, could affect the prognosis of different cancer types. As expected, we discovered that the prognosis of 8 different cancer types was correlated with pyroptosis activity. However, the correlations are divergent, as pyroptosis positively correlated with patient survival in some cancer types while negatively associated with patient survival in others, which we labeled as PPRC and PNRC (Figures 2(a)–2(d)). Notably, later identified hub genes are not directly involved in pyroptosis signaling per se, so there should be unexploited mechanisms linking them with pyroptosis-related cancer prognosis, possibly by tumor immunity related biological processes (Figures 3(c) and 3(d)). Indeed, *π* signature is differentially correlated with immune cell infiltration in two cancer subtypes (Figure 5(a)), and the substantial correlations with the TIDE score suggest that this signature could be used as a predictor tool for immunotherapy responses (Figures 5(d) and 5(g)). These findings suggested that the two batches of PPRC and PNRC signature genes might reflect different tumor immunity responses in different pyroptosis-related cancer subtypes, and the signature was further validated as a prognostic marker and a pathological stage indicator in patients from several public cancer databases (Figures 4(a)–4(d)), demonstrating its prognostic and predictive relevance in pyroptosis-related cancer subtypes.

Importantly, the *π* signature is also linked to other important predictive and prognostic molecules such as immune checkpoint molecules, traditional oncogenes, and tumor-suppressor genes. Oncogene and tumor-suppressor gene somatic mutations play a crucial role in carcinogenesis, as they are linked to unchecked proliferation and immune evasion. Oncogenic mutations in TP53, KRAS, and IDH1 contribute to tumorigenesis, but their role in patient prognosis is still unclear. For example, IDH mutation plays an important role in the progression of early stage of gliomas [33], but it is beneficial for younger patients and associated with a better prognosis when compared to patients with wild-type IDH1 [34]. KRAS and TP53 mutations are carcinogenesis drivers that have been linked to poor patient outcomes in a variety of cancers [35, 36]. However, studies also found that TP53-mutated glioma patients under the age of 70 have a better prognosis than TP53 wild-type glioma patients [37]. In our study, patients with GRIN2A mutations in SKCM and IDH1, TP53, and ATRX mutations in GBMLGG have a better prognosis than wild-type patients, whereas patients with KRAS and TP53 mutations in PAAD have a worse prognosis (Supplementary Figure 7), and the *π* signature correlated positively or negatively with several oncogenic molecules (Figure 4(e)). Thus, our data provided a novel sight that these mutations might affect tumor progression and patients' prognosis through pyroptosis-related mechanisms. Immune checkpoint molecules such as CTLA-4, PD-1, and PD-L1 have been validated as targets in cancer immunotherapy, but few studies have explored their roles in cancer prognosis [38–40]. We discovered that the expression of immune checkpoint molecules correlated with *π* signature differentially between the cancer subtypes (Figures 5(c) and 5(d)). It should be noted that PD-L1 has been proven to be associated with pyroptosis through upregulation of GSDMC [18], and since our study found additional immune checkpoint molecules have strong correlations with pyroptosis as well, further studies are required in this area. These findings show that *π* signature can be used as a precise tool for cancer type-specific detection and prognosis at the molecular level.

Pyroptosis, which could be induced by chemotherapy, is directly involved in tumor cell viability and indirectly involved in tumor immunity and TME modulation via DAMPs, so it is critical to assess chemotherapy drug responses in various cancers prior to pyroptosis-based intervention or combinational therapy. We successfully established a predicting model with the remarkable correlations between the *π* signature and drug IC50 values in each cancer subtype (Figures 6(a) and 6(b)). In this model, we provided a list of potentially effective drugs for patients with different scores in cancer subtypes (Table 3), providing a new way to improve the efficacy of chemotherapy or combined immunotherapy. Finally, we created nomogram models in which *π* signature was used as an independent predictor of OS in PPRC and PNRC patients (Supplementary Figure 10A-B), further consolidating the prognostic value of *π* signature.

Several studies have shown that the pyroptosis core genes behave differently in different tumors, with GSDMC, GSDMD, and GSDMB acting as oncogenes in colorectal cancer, small-cell lung cancer, and breast cancer, respectively [41–43], while GSDME acts as a tumor suppressor in a variety of cancers [15, 44, 45]. In other investigations, GSDMA, GSDMB, GSDMC, and GSDMD have also been shown to have tumor-suppressing properties [14, 46, 47]. Thus, diagnosis or prognosis by a single pyroptosis gene is unreliable. Several reports also identified pyroptosis-related multiple-gene signatures for specific cancers, with the majority of these studies focusing on 4–9 pyroptosis core genes to construct prognostic models [28, 48–50]. In contrast to these studies, we divided pyroptosis-related cancer types into subgroups, exploited and elaborated hub genes found in distinct genetic backgrounds, and expanded signature genes to include tumor immunity-related biological processes but not those directly engaged in pyroptosis. Despite the fact that the pathways linking those signature genes to pyroptosis have not been experimentally tested, it may reveal the underlying links between pyroptosis-related tumor immunity and disease progression or prognosis. In summary, we identified the *π* signature in various pyroptosis-related cancer subtypes as a reliable prognostic and predictive tool that could be both useful in mechanistic and clinical studies.

## 4. Materials and Methods

### 4.1. DNA and RNA Sequencing Data of Pan-Cancer Samples

All cohort datasets included in this study were summarized in Table 1. Transcriptional mRNA data, somatic mutations, and clinical data including age, gender, tumor stage, pathology stage, and OS of training cohorts were obtained from TCGA database. All 5 validation cohorts containing mRNA expression data and overall survival were downloaded from the Chinese Glioma Genome Atlas (CGGA) database, RECA-EU (International Cancer Genome Consortium, ICGC), and Gene Expression Omnibus (GEO) database under accession numbers GSE42568, GSE65904, and GSE78220. Patients without OS information were removed from our study.

### 4.2. Calculations of PCD Scores, TMB, and Gene Mutation Frequency

The pyroptosis core gene list was determined as described in the introduction section. Gene lists for apoptosis and autophagy were obtained from literature reviews [51, 52]. Single sample gene set enrichment analysis (ssGSEA) was applied to calculate PCD scores using [53]. TMB for each tumor tissue can be calculated by the R package “maftools” using the VarScan method [54]. The R package “maftools” was also used to calculate the mutation frequencies of genes from PCD gene lists in somatic mutation analysis.

### 4.3. Identification of the Hub Genes of Pyroptosis Positively Related Cancer (PPRC) and Pyroptosis Negatively Related Cancer (PNRC)

The DEGs between the high and low pyroptosis score groups across pan-cancer types were identified using the R package “limma” [55]. DEGs were defined using a *P* value less than 0.05 and an absolute log  2 fold change larger than 1. BP analysis of GO was used to investigate the biological role of hub genes. Significantly enriched BPs were defined as those with a *P* value less than 0.05.

### 4.4. Construction and Validation of *π* Signature Models

Univariate Cox regression analysis and the Cox proportional hazard model were adopted for the construction of the optimal gene set from hub genes using R packages “survival” and “glmnet” [56]. The linear combination of gene expression weighted by regression coefficients (coeffs) was established to generate each patient's PP score or PN score with the following formula:

(1) $$\begin{array}{c} \text{PP}-\text{Score}=\sum_{a=1}^{n}\left({\text{Coeff}}_{a}\times {\text{Exp}}_{a}\right),\\ \text{PN}-\text{Score }=\sum_{b=1}^{n}\left({\text{Coeff}}_{b}\times {\text{Exp}}_{b}\right), \end{array}$$

where “*a*” stood for the 11 selected PPRC hub genes, “*b*” for the 39 selected PNRC hub genes, and “Exp” for the mRNA expression level. These signatures were termed “*π*” signature (the onset syllable of pyroptosis), and the PP score and PN score together were termed *π* score. The optimal cutoff values for *π* score were determined by the R package “survminer.” In addition, the R package “survivalROC” was used to develop time-dependent ROC curves with AUC values in order to assess the predictive efficacy of the signature genes.

### 4.5. Association Analysis between the *π* Signature and Clinical Features

The tumor stages, pathology M (distant organ metastasis), and pathology N (lymph node metastasis) stage information were obtained alongside the transcriptome data in the TCGA database. The tumor stages of GBM and LGG were not accessible, so we considered GBM patients as III/IV stages and LGG patients as I/II stages. The association between the pyroptosis score or *π* score and tumor stages and pathology M, and pathology N stages was investigated using Student's *t*-test, with a *P* value < 0.05 considered statistically significant.

### 4.6. Immune Cell Infiltration Analysis and Immunotherapy Response Prediction

XCELL was used to estimate the immune cell composition of patients with each cancer type [57]. The immune score, stromal score, and TME score of patients were also calculated by XCELL. Potential immune checkpoint blockade responses were predicted with the Tumor Immune Dysfunction and Exclusion (TIDE) algorithm, which is a computational framework that identifies factors underlying tumor immune escape by multiple published transcriptomic biomarkers [58, 59].

### 4.7. Correlation Study of *π* Signature and Drug Sensitivity

Chemotherapy responses for each patient were predicted based on two public pharmacogenomics databases: Genomics of Drug Sensitivity in Cancer (GDSC) and the Broad Institute's Cancer Therapeutics Response Portal (CTRP). The drug sensitivities of each sample were estimated using the R package “oncoPredict” which enabled the generation of anticipated drug response models that could be used as a virtual screen for patient drug response [60]. Then, using Pearson analysis, we investigated the association between the *π* signature and drug sensitivity. Signaling pathways targeted by the top 20 correlated drugs were analyzed in different cancer subtypes.

### 4.8. Construction of Nomogram Models for Patient OS Prediction

Univariable Cox regression analysis was performed to select independent clinicopathologically prognostic factors for OS prediction, and as a result, cancer type, *π* signature, as well as clinical characteristics considering age and tumor stages were selected to construct two visualized prognostic nomogram models. For patients from various cancer subtypes, the R packages “rms” and “survival” were used to predict the probability of 1-, 3-, and 5-year overall survival. The predictability of the nomogram models was assessed using a bootstrap approach with 1,000 resamplings to measure discrimination and calibration. Discrimination was accessed via the C-index, which is used to evaluate the predictive value of a nomogram. When the C-index is closer to 1, it implies a more accurate predictive ability of the nomogram. The calibration curves evaluated the consistency between the nomogram's predicted and actual survival probabilities.

### 4.9. Statistical Analysis

Data were analyzed using R 3.5.2 (https://www.r-project.org/) and GraphPad Prism 8.0 software. We used the D'Agostino and Pearson test to test data for normality. Student's *t* test or Kruskal–Wallis one-way ANOVA were used to determine the relationship between the pyroptosis score or *π* score and tumor stages, gene mutations, and the TIDE score, with a *P* value of 0.05 considered statistically significant. (Student's *t* test is used to compare the means between the two groups, whereas ANOVA is used to compare the means among three or more groups.) The Pearson correlation method was used for correlation analysis. The Kaplan–Meier method was used to calculate the survival probability in terms of OS, and the log-rank test was used to examine intergroup differences. Univariate, multivariate analyses, and lasso regression were performed through a Cox proportional hazard model, and *P* value < 0.05 was considered as the statistical significance.

## Figures and Tables

**Table 1:** Tumor cohort datasets included in this study.

	Data source	Cancer type	Number of samples
*Training cohort*			
BRCA	The Cancer Genome Atlas (TCGA)	Breast cancer	1059
GBM	The Cancer Genome Atlas (TCGA)	Glioblastoma	167
KIRC	The Cancer Genome Atlas (TCGA)	Kidney clear cell carcinoma	528
LGG	The Cancer Genome Atlas (TCGA)	Lower grade glioma	524
MESO	The Cancer Genome Atlas (TCGA)	Mesothelioma	84
PAAD	The Cancer Genome Atlas (TCGA)	Pancreatic cancer	174
SKCM	The Cancer Genome Atlas (TCGA)	Melanoma	406
UVM	The Cancer Genome Atlas (TCGA)	Ocular melanoma	79

*Validation cohort*			
CGGA	Chinese Glioma Genome Atlas (CGGA)	Glioma	657
RECA-EU	International Cancer Genome Consortium (ICGC)	Kidney clear cell carcinoma	91
GSE42568	Gene Expression Omnibus (GEO)	Breast cancer	104
GSE65904	Gene Expression Omnibus (GEO)	Melanoma	210
GSE78220	Gene Expression Omnibus (GEO)	Melanoma	26

**Table 2:** Comparison between *π* signature and the other previously established pyroptosis-related signatures.

Cancer subtype	Signatures	Cancer types	AUC at 1 year	AUC at 3 years	AUC at 5 years	References
PPRC	*π* signature	BRCA, MESO, and SKCM	0.787	0.774	0.748	
	Other signatures	Breast cancer (BRCA)	0.756	0.752	0.723	[27]
		Skin cutaneous melanoma (SKCM)	—	0.64	0.711	[28]

PNRC	*π* signature	GBM, KIRC, LGG, PAAD, and UVM	0.844	0.861	0.829	
	Other signatures	Glioma (GBM and LGG)	0.669	0.713	0.709	[29]
		Kidney renal clear cell carcinoma (KIRC)	0.57	0.62	0.65	[30]
		Pancreatic adenocarcinoma (PAAD)	0.596	0.687	0.732	[31]
		Uveal melanoma (UVM)	0.79	0.854	0.886	[32]

**Table 3:** Potential chemotherapy drugs recommended based on the *R* correlation between the *π* score and the drug sensitivity.

	Positively correlated drug	*R*	FDR	Negatively correlated drug	*R*	FDR
BRCA	Ruxolitinib	0.388	<0.0001	SB505124	−0.179	<0.0001
	OF-1	0.383	<0.0001	ERK_2440	−0.104	0.0007
	Gallibiscoquinazole	0.367	<0.0001	BI-2536	−0.084	0.0062
	MN-64	0.363	<0.0001	Sepantronium bromide	−0.067	0.0299
	KRAS (G12C) inhibitor-12	0.362	<0.0001			

MESO	AZD8055	0.675	<0.0001	SB505124	−0.338	0.0017
	KU-55933	0.593	<0.0001	Dihydrorotenone	−0.301	0.0055
	Telomerase inhibitor IX	0.564	<0.0001			
	MIM1	0.544	<0.0001			
	Bortezomib	0.541	<0.0001			

SKCM	SB216763	0.611	<0.0001	ERK_6604	−0.299	<0.0001
	AZD8055	0.582	<0.0001	ERK_2440	−0.295	<0.0001
	AMG-319	0.577	<0.0001	SCH772984	−0.230	<0.0001
	PRIMA 1MET	0.571	<0.0001	Trametinib	−0.220	<0.0001
	GSK591	0.558	<0.0001	SB505124	−0.197	<0.0001

GBM	Vorinostat	0.401	<0.0001	Entospletinib	−0.413	<0.0001
	ABT737	0.393	<0.0001	ERK_2440	−0.403	<0.0001
	WEHI 539	0.350	<0.0001	SCH772984	−0.365	<0.0001
	Tamoxifen	0.324	<0.0001	Dasatinib	−0.355	<0.0001
	TAF1_5496	0.280	0.0003	ULK1_4989	−0.333	<0.0001

KIRC	SB505124	0.476	<0.0001	AGI 5198	−0.395	<0.0001
	Erlotinib	0.291	<0.0001	XAV939	−0.354	<0.0001
	OF-1	0.286	<0.0001	AZD8055	−0.351	<0.0001
	BI-2536	0.261	<0.0001	ULK1_4989	−0.324	<0.0001
	IAP_5620	0.253	<0.0001	Topotecan	−0.318	<0.0001

LGG	NVP-ADW742	0.533	<0.0001	KU 55933	−0.460	<0.0001
	SB505124	0.512	<0.0001	Entospletinib	−0.444	<0.0001
	Vorinostat	0.508	<0.0001	XAV939	−0.421	<0.0001
	Linsitinib	0.473	<0.0001	AZD1332	−0.419	<0.0001
	Lapatinib	0.421	<0.0001	AZD8055	−0.405	<0.0001

PAAD	Vorinostat	0.442	<0.0001	SCH772984	−0.499	<0.0001
	BIBR-1532	0.427	<0.0001	Acetalax	−0.492	<0.0001
	Doramapimod	0.424	<0.0001	ERK_6604	−0.484	<0.0001
	Sorafenib	0.417	<0.0001	Sapitinib	−0.480	<0.0001
	TAF1_5496	0.402	<0.0001	Trametinib	−0.459	<0.0001

UVM	Sepantronium bromide	0.306	0.0061	XAV939	−0.235	0.0369
	Docetaxel	0.293	0.0088			
	Epirubicin	0.292	0.0091			
	SB505124	0.287	0.0104			
	Sinularin	0.286	0.0105			

## Data Availability

The data used are obtained from TCGA data portal: https://portal.gdc.cancer.gov/, the CGGA database: https://www.cgga.org.cn/, the ICGC database: https://dcc.icgc.org/, and GEO datasets: https://www.ncbi.nlm.nih.gov/gds/.

## Supplementary Materials

Supplementary Materials Supplementary Figure 1: calculation of apoptosis, autophagy, and pyroptosis scores in cancer patients. Supplementary Figure 2: heatmaps showed the expression of 13 pyroptosis core genes in 8 cancer types. Supplementary Figure 3: volcano plots revealed up- (red) and down-regulated (blue) DEGs between the high and low pyroptosis score groups of patients in each cancer type. Supplementary Figure 4: hub genes in PPRC and PNRC. Supplementary Figure 5: Lasso Cox analyses of the signature score model of PPRC and PNRC. Supplementary Figure 6: KM analyses of patients with high or low *π* scores. Supplementary Figure 7: Corrplot of the correlation between the expression of 13 pyroptosis core genes and the *π* score. Supplementary Figure 8: KM survival curves of the top 3 mutated genes from 8 cancer types in TCGA cohort. Supplementary Figure 9: The development of the predictive PPRC and PNRC nomogram models. Supplementary Figure 10: The development of the nomogram models predicting the patients' overall survival. Supplementary Table 1: cutoff values of pyroptsosis scores from 8 different cancer types. Supplementary Table 2: identification of hub genes of PPRC and PNRC. Supplementary Table 3: GO enrichment analysis of PPRC hub genes. Supplementary Table 4: GO enrichment analysis of PNRC hub genes. Supplementary Table 5: univariate Cox regression analysis of hub genes from PPRC. Supplementary Table 6: univariate Cox regression analysis of hub genes from PNRC. Supplementary Table 7: multivariate regression analysis of OS-related hub genes from PPRC. Supplementary Table 8: multivariate regression analysis of OS-related hub genes from PNRC. Supplementary Table 9: cutoff values of PP or PN scores from 8 different cancers. Supplementary Table 10: top 3 mutated genes of each cancer type. Supplementary Table 11: correlation between the *π* score and IC50 of 198 chemotherapy drugs.
