# Supplementary material for: Pan-Cancer Pyroptosis Analyses Identified Novel Immunology and Chemotherapy-Related Prognostic Signatures in Cancer Subtypes
Source: J Oncol. 2022 Jun 20;2022:6609297. doi: 10.1155/2022/6609297 (PMC9236821; doi:10.1155/2022/6609297)
Supplement: Supplementary Materials — Supplementary Figure 1: calculation of apoptosis, autophagy, and pyroptosis scores in cancer patients. Supplementary Figure 2: heatmaps showed the expression of 13 pyroptosis core genes in 8 cancer types. Supplementary Figure 3: volcano plots revealed up- (red) and down-regulated (blue) DEGs between the high and low pyroptosis score groups of patients in each cancer type. Supplementary Figure 4: hub genes in PPRC and PNRC. Supplementary Figure 5: Lasso Cox analyses of the signature score model of PPRC and PNRC. Supplementary Figure 6: KM analyses of patients with high or low π scores. Supplementary Figure 7: Corrplot of the correlation between the expression of 13 pyroptosis core genes and the π score. Supplementary Figure 8: KM survival curves of the top 3 mutated genes from 8 cancer types in TCGA cohort. Supplementary Figure 9: The development of the predictive PPRC and PNRC nomogram models. Supplementary Figure 10: The development of the nomogram models predicting the patients' overall survival. Supplementary Table 1: cutoff values of pyroptsosis scores from 8 different cancer types. Supplementary Table 2: identification of hub genes of PPRC and PNRC. Supplementary Table 3: GO enrichment analysis of PPRC hub genes. Supplementary Table 4: GO enrichment analysis of PNRC hub genes. Supplementary Table 5: univariate Cox regression analysis of hub genes from PPRC. Supplementary Table 6: univariate Cox regression analysis of hub genes from PNRC. Supplementary Table 7: multivariate regression analysis of OS-related hub genes from PPRC. Supplementary Table 8: multivariate regression analysis of OS-related hub genes from PNRC. Supplementary Table 9: cutoff values of PP or PN scores from 8 different cancers. Supplementary Table 10: top 3 mutated genes of each cancer type. Supplementary Table 11: correlation between the π score and IC50 of 198 chemotherapy drugs. [file 6609297.f1.zip › 6609297.f1/Supplementary Figures.PDF]

Figure S1

A

Apoptosis			Autophagy			Pyroptosis		
AKT1	BIRC7	MAP2K1	AMBRA1	ATG4D	MTOR	CASP1	GSDMA	GSDME
APAF1	CASP3	MAPK1	ATG101	ATG5	PIK3C3	CASP3	GSDMB	GZMA
BAD	CASP6	MCL1	ATG12	BCL2	PIK3R4	CASP4	GSDMC	GZMB
BAK1	CASP7	MTOR	ATG13	BECN1	RALB	CASP5	GSDMD	PJVK
BAX	CASP8	MYC	ATG14	EXOC8	RB1CC1	CASP8		
BCL2	CASP9	NFKB1	ATG16L1	GABARAPL1	ULK1			
BCL2L1	DIABLO	NFKB2	ATG4A	MAP1LC3A	WIPI1			
BIRC2	FADD	PIK3CA	ATG4B	MAP1LC3B	WIPI2			
BIRC3	FASLG	RIPK1	ATG4C	MAP1LC3C	ZFYVE1			
BIRC5	KRAS	TNF						

B

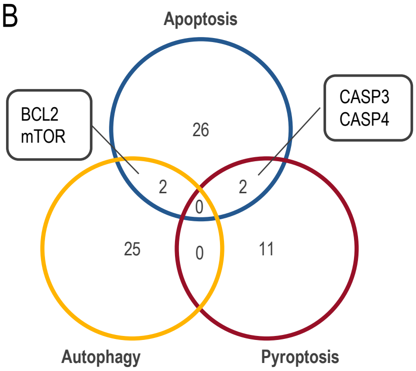

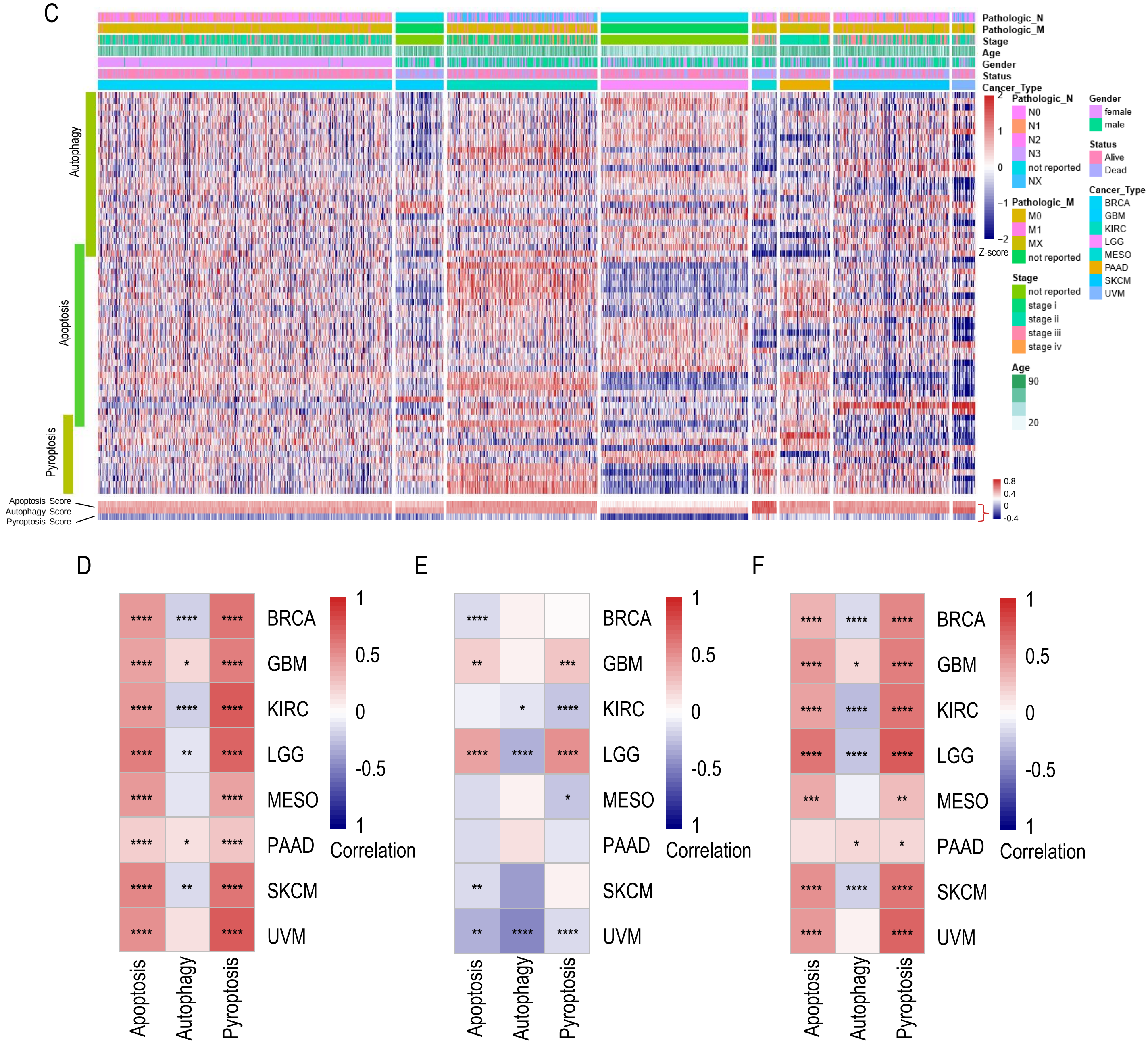

Supplementary Figure 1. Calculation of apoptosis, autophagy, and pyroptosis scores in cancer patients.

(A) Gene lists selected to calculate the apoptosis, autophagy, and pyroptosis scores. (B) Venn plot of genes selected for 3 PCD scores calculation. (C) Heatmap showed PCD-related gene expression and 3 PCD scores in different cancer patients. (D-F) Heatmaps showed the Pearson correlation coefficients (R) between pyroptosis score and Immune score (D), pyroptosis score and Stromal score (E), pyroptosis score and TME score (F). $^{*}P < 0.05$, $^{**}P < 0.01$, $^{***}P < 0.001$, $^{****}P < 0.0001$.

Figure S2

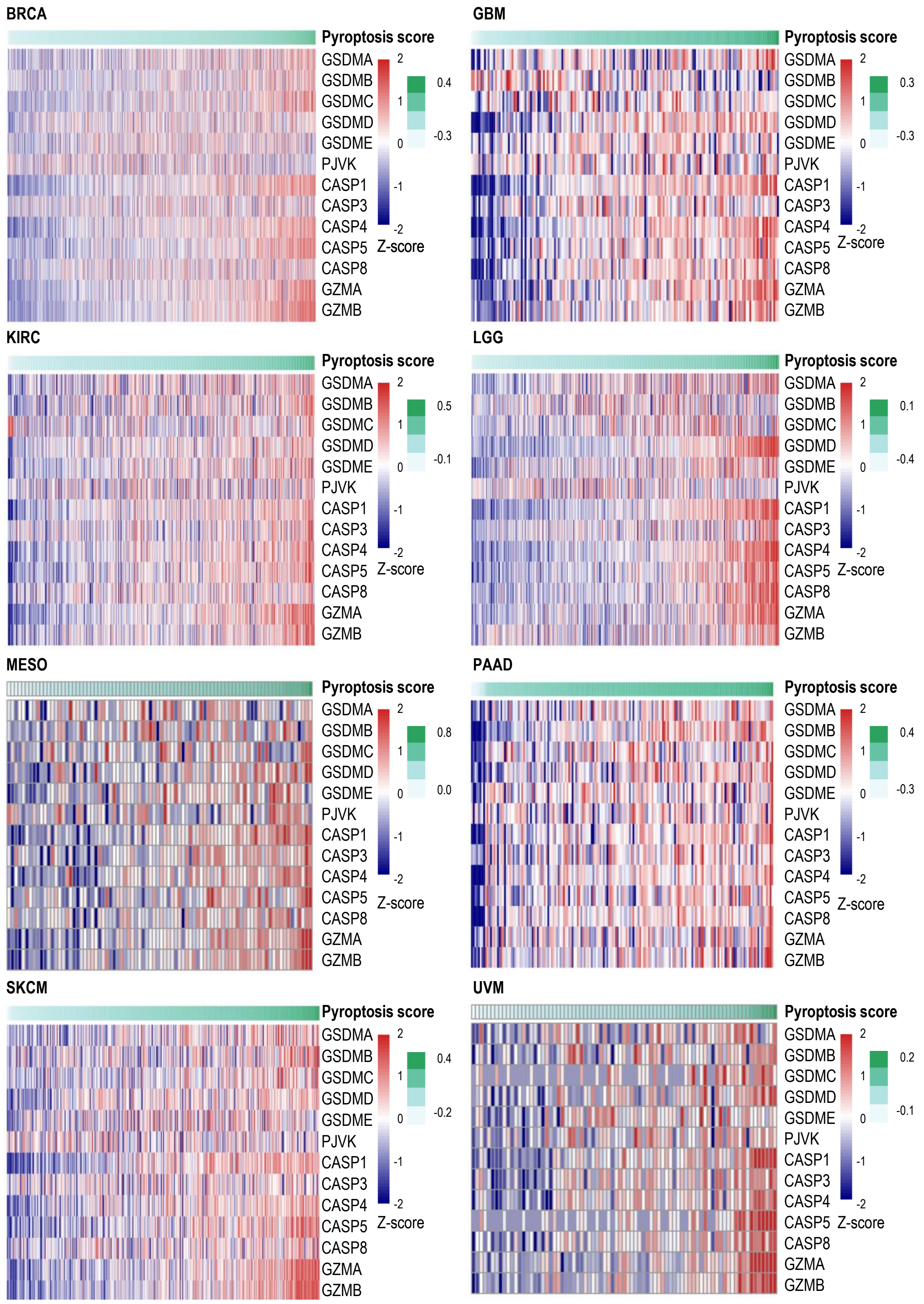

Supplementary Figure 2. Heatmaps showed the expression of 13 pyroptosis core genes in 8 cancer types.

Figure S3

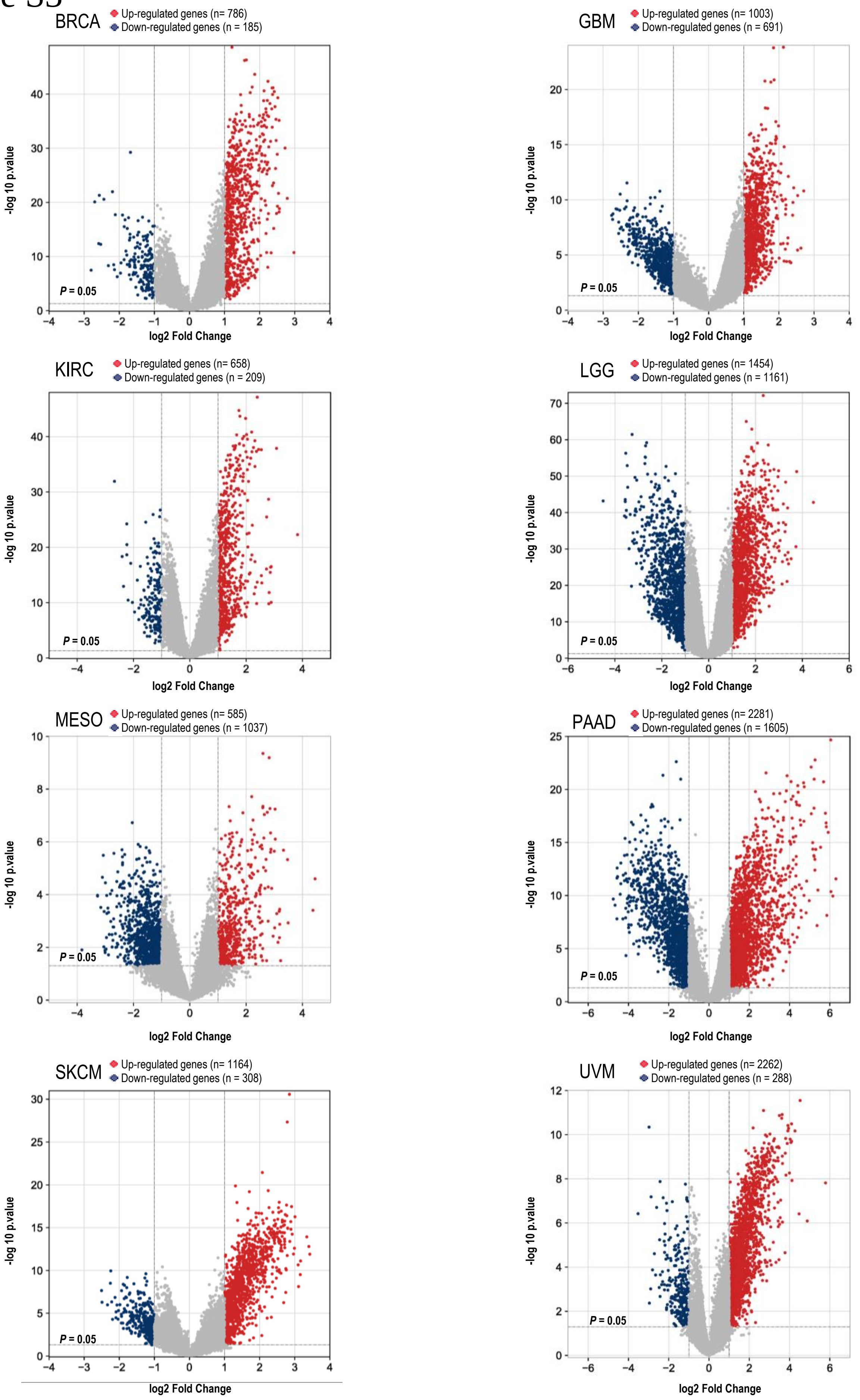

Supplementary Figure 3. Volcano plots revealed up- (red) and down-regulated (blue) DEGs between high and low pyroptosis score groups of patients in each cancer type.

Figure S4

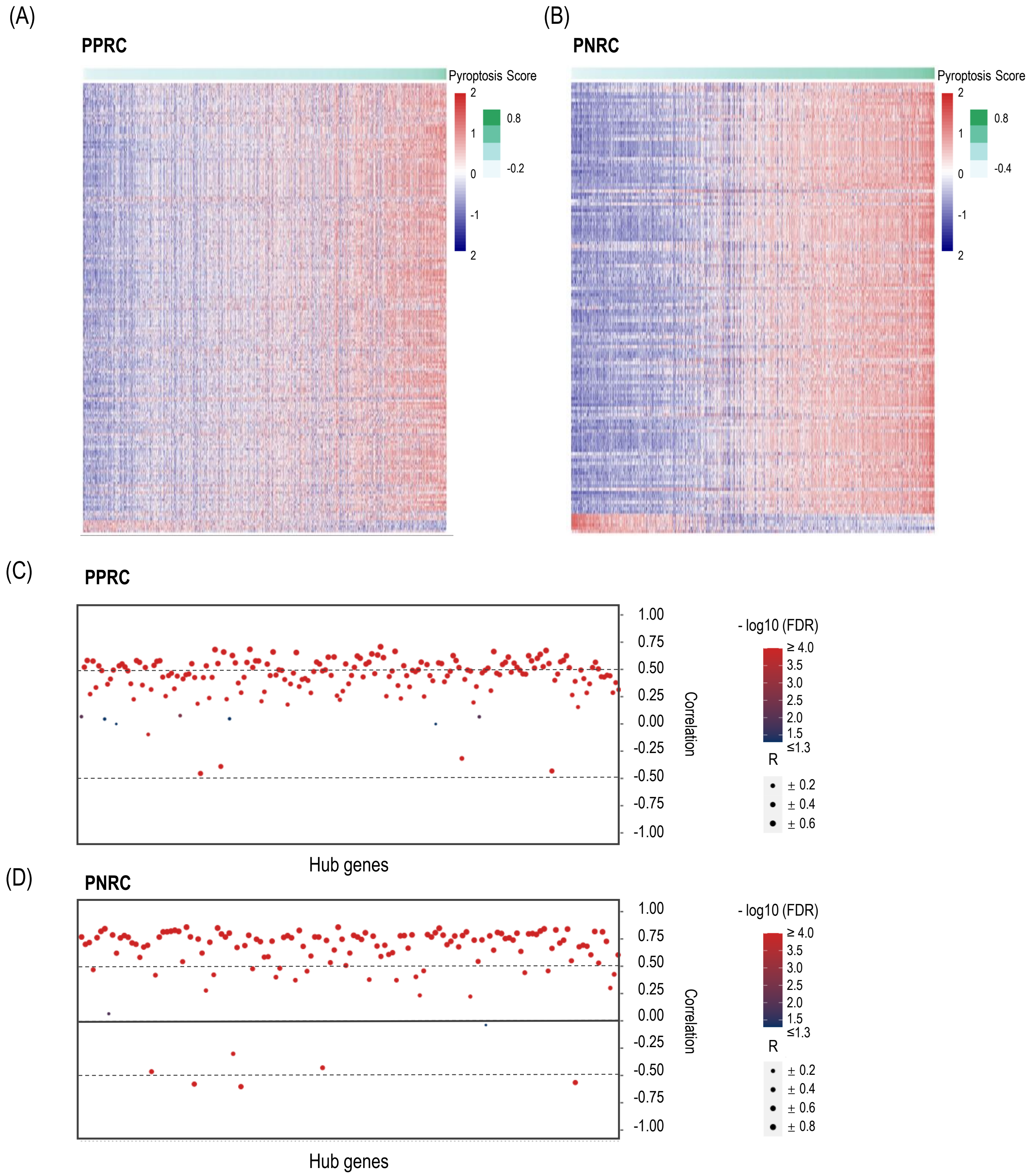

Supplementary Figure 4. Hub genes in PPRC and PNRC.
(A-B) Heatmaps showed the expression of 186 hub genes in PPRC (A) and 139 hub genes in PNRC (B). The gene symbols for the hub genes were not shown. (C-D) Dot plots showed the correlation between the expression of hub genes and pyroptosis score (C-D). The gene symbols for the hub genes were not shown.

Figure S5

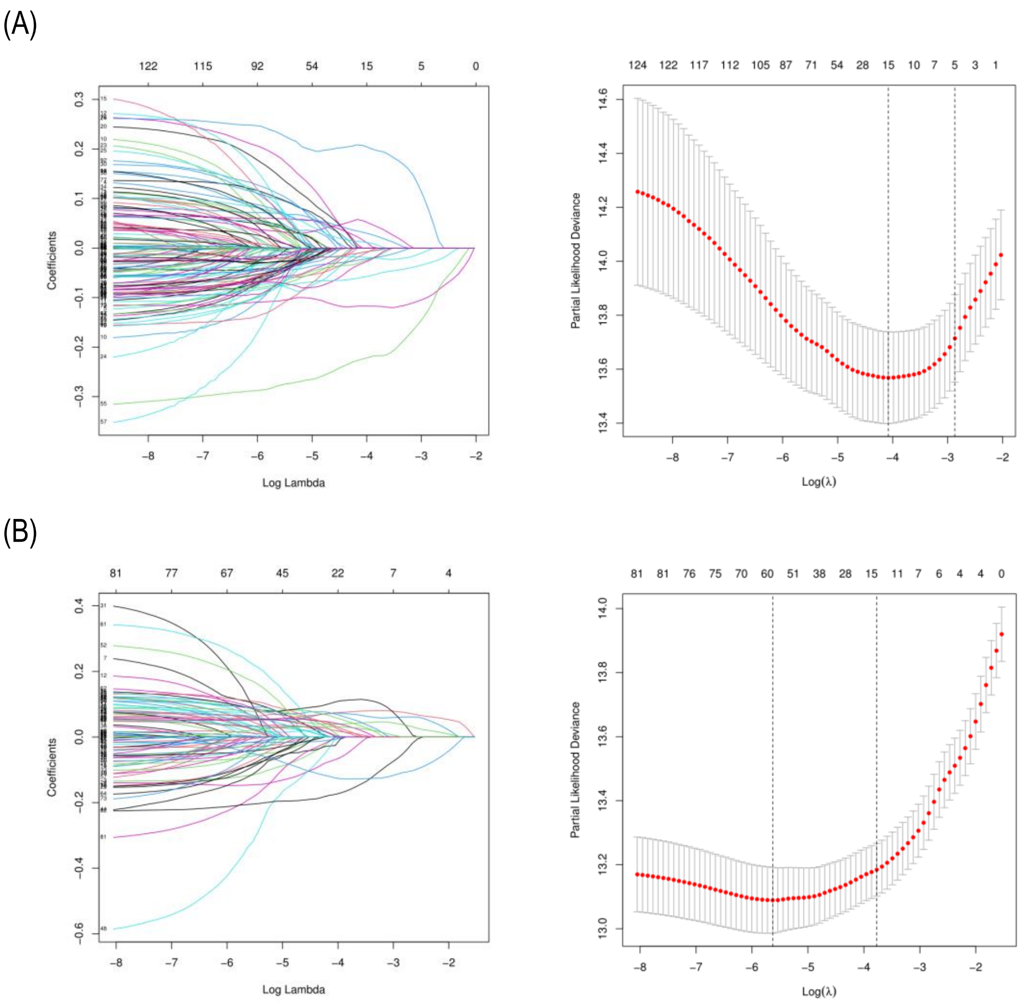

Supplementary Figure 5. Lasso Cox analyses of the signature score model of PPRC and PNRC.

(A-B) Lasso Cox regression models were constructed from the 132 OS-related hub genes from PPRC (A) and 85 from PNRC (B). The tuning parameters λ were calculated based on the partial likelihood of deviance with 10-fold cross-validation, and the coefficients were plotted against log (λ). An 11-gene signature for PPRC (A) and another 39-gene signature for PNRC (B) were identified according to the best-fit profiles.

Figure S6

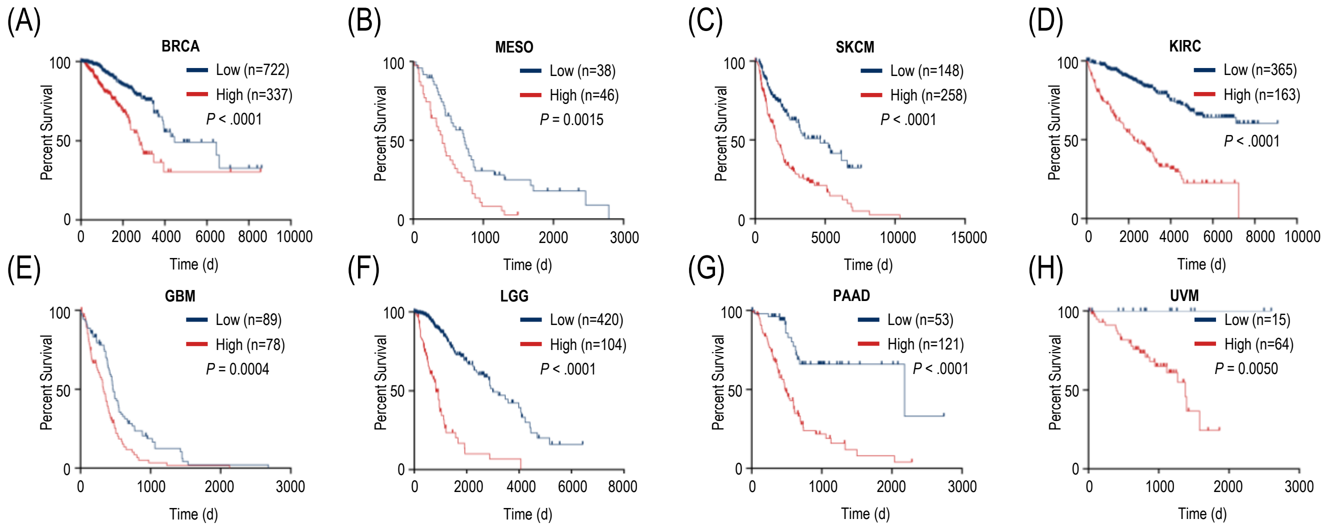

Supplementary Figure 6. KM analyses of patients with high or low π scores
(A-C) Survival curves showed high PP-Score was associated with poor prognosis in BRCA (A), MESO (B), and SKCM (C). Survival curves were compared using the log-rank test. High (red) and low (blue) PP-Scores were determined by the R package "Survminer". (D-H) Survival curves showed high PN-Score was associated with poor prognosis in KIRC (D), GBM (E), LGG (F), PAAD (G), and UVM (H). Survival curves were compared using the log-rank test. High (red) and low (blue) PN-Scores were determined by the R package "Survminer".

Figure S7

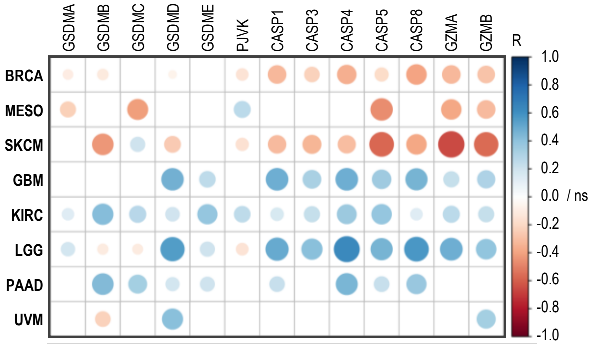

Supplementary Figure 7. Corrplot of the correlation between the expression of 13 pyroptosis core genes and π score. R, Pearson correlation coefficients; ns, not significant.

Figure S8

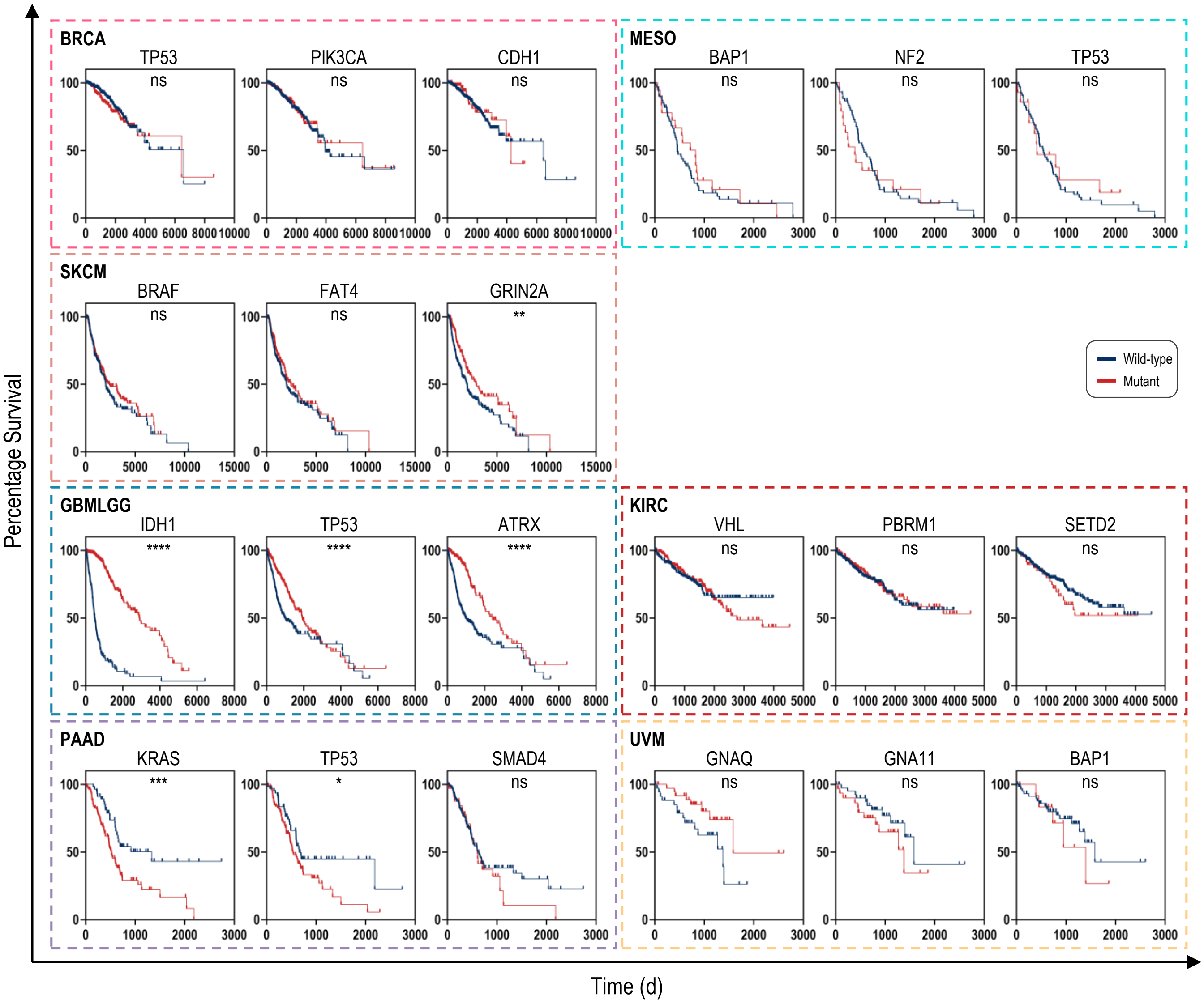

Supplementary Figure 8. KM survival curves of the top 3 mutated genes from 8 cancer types in the TCGA cohort. Red stood for gene mutated patients, and blue stood for wild-type patients. Survival curves were compared using the log-rank test. *P < 0.05, **P < 0.01 , ***P < 0.001, ****P < 0.0001; ns, not significant.

Figure S9

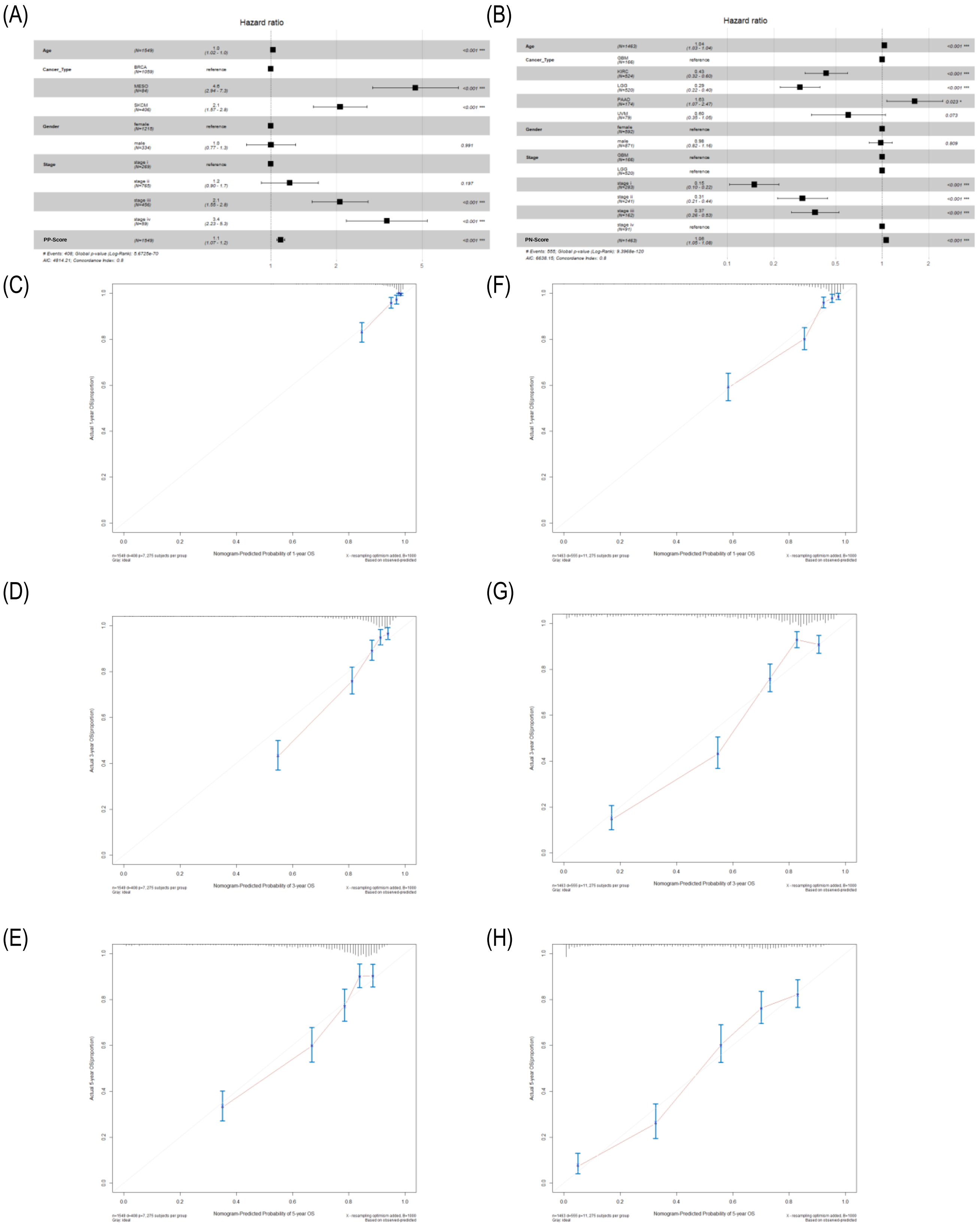

Figure S9. Development of the predictive PPRC and PNRC nomogram models.
(A-B) Forest plots of the multivariate Cox regression analyses indicated the PP-Score model (A) and PN-Score model (B) were independent predictors for OS. (C-D) Calibration curves of the PPRC nomogram model concerning consistency between predicted and actual 1-year (C), 3-year (D), and 5-year (E) OS. (F-H) Calibration curves of the PNRC nomogram model concerning consistency between predicted and actual 1-year (F), 3-year (G), and 5-year (H) OS.

Figure S10

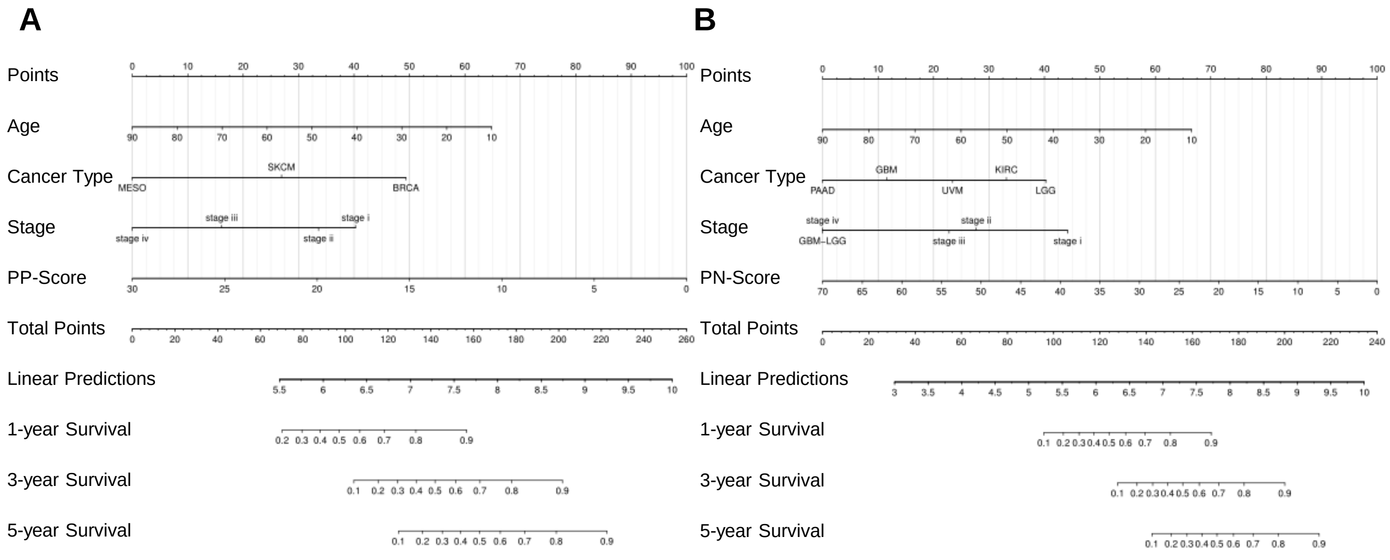

Figure S10. Development of the nomogram models predicting the patients' overall survival
(A-B) Nomogram models for predicting the probability of 1-year, 2-year, and 3-year OS in PPRC patients (A) and in PNRC patients (B). Score of each parameter was identified through plotting a straight-line upwards and crossing with the points line. The total points of each patient is the sum of each parameter score crossed with the points line. The patients' survival rates in 1, 3, and 5 years were estimated by plotting a perpendicular line downwards from the total point axis to the resulting axis scaled with probability. The concordance index (C-index) was 0.800 (95% CI: 0.760-0.840) for the PPRC nomogram model (A) and 0.803 (95% CI: 0.762-0.843) for the PNRC nomogram model (B).
