# Supplementary material for: Pan-Cancer Pyroptosis Analyses Identified Novel Immunology and Chemotherapy-Related Prognostic Signatures in Cancer Subtypes
Source: J Oncol. 2022 Jun 20;2022:6609297. doi: 10.1155/2022/6609297 (PMC9236821; doi:10.1155/2022/6609297)
Supplement: Supplementary Materials — Supplementary Figure 1: calculation of apoptosis, autophagy, and pyroptosis scores in cancer patients. Supplementary Figure 2: heatmaps showed the expression of 13 pyroptosis core genes in 8 cancer types. Supplementary Figure 3: volcano plots revealed up- (red) and down-regulated (blue) DEGs between the high and low pyroptosis score groups of patients in each cancer type. Supplementary Figure 4: hub genes in PPRC and PNRC. Supplementary Figure 5: Lasso Cox analyses of the signature score model of PPRC and PNRC. Supplementary Figure 6: KM analyses of patients with high or low π scores. Supplementary Figure 7: Corrplot of the correlation between the expression of 13 pyroptosis core genes and the π score. Supplementary Figure 8: KM survival curves of the top 3 mutated genes from 8 cancer types in TCGA cohort. Supplementary Figure 9: The development of the predictive PPRC and PNRC nomogram models. Supplementary Figure 10: The development of the nomogram models predicting the patients' overall survival. Supplementary Table 1: cutoff values of pyroptsosis scores from 8 different cancer types. Supplementary Table 2: identification of hub genes of PPRC and PNRC. Supplementary Table 3: GO enrichment analysis of PPRC hub genes. Supplementary Table 4: GO enrichment analysis of PNRC hub genes. Supplementary Table 5: univariate Cox regression analysis of hub genes from PPRC. Supplementary Table 6: univariate Cox regression analysis of hub genes from PNRC. Supplementary Table 7: multivariate regression analysis of OS-related hub genes from PPRC. Supplementary Table 8: multivariate regression analysis of OS-related hub genes from PNRC. Supplementary Table 9: cutoff values of PP or PN scores from 8 different cancers. Supplementary Table 10: top 3 mutated genes of each cancer type. Supplementary Table 11: correlation between the π score and IC50 of 198 chemotherapy drugs. [file 6609297.f1.zip › 6609297.f1/Supplementary Tables.pdf]

Table S1: Cutoff values of pyroptsosis scores from 8 different cancer types

Cancer types	Cutoff values	Number of patients	
		Low	High
BRCA	-0.114	108	951
GBM	-0.123	34	133
KIRC	0.034	134	394
LGG	-0.120	425	99
MESO	0.478	74	10
PAAD	0.020	16	158
SKCM	-0.069	50	356
UVM	0.020	60	19

Table S2: Identification of hub genes of PPRC and PNRC

PPRC					
ACSL5	CCL7	DTHD1	IFI44	NCR3	SLA2
ADAMDEC1	CCL8	EPSTI1	IFI44L	NKG7	SLC15A3
ANKRD22	CCR5	ETV7	IFI6	NLRC5	SMTNL1
AOAH	CD2	F5	IFNG	NUGGC	SP140
APBB1IP	CD247	FASLG	IKZF3	OAS2	STAP1
APOBEC3A	CD274	FCN1	IL12RB1	OASL	TAP1
APOBEC3D	CD38	FCRL6	IL15RA	PATL2	TBX21
APOBEC3G	CD3D	GBP1	IL2RB	PDCD1	TFPI2
APOBEC3H	CD3E	GBP2	IL2RG	PDZK1IP1	THEMIS
APOL1	CD3G	GBP4	IL4I1	PLAAT4	TIFAB
APOL3	CD48	GBP5	IRF1	PRF1	TIGIT
APOL6	CD7	GBP6	ISG15	PSMB8	TMEM150B
AQP9	CD80	GIMAP2	ITGAL	PSMB9	TMIGD2
B2M	CD8A	GNLY	ITGB7	PTCRA	TMPRSS3
BCL2L14	CD8B	GPR171	JAKMIP1	PTGDR	TNFSF13B
BIRC3	CD96	GPR18	KCNJ10	PYHIN1	TRABD2A
BST2	CFB	GPR31	KIR2DL4	RARRES1	TRIM22
BTN3A3	CHI3L1	GZMA	KIR3DL2	RSAD2	TTC22
C1QA	CHIT1	GZMB	KLHDC7B	RTP4	TYMP
C1QB	CLEC4D	GZMH	KLK10	RTP5	UBASH3A
C1QC	CLEC4E	GZMK	KLRB1	RUFY4	VNN3
C1R	CLIC3	HCST	KLRC1	S100A8	VSIR
C1S	CRTAM	HLA-A	KLRD1	S100A9	XAF1
CALHM6	CST7	HLA-B	KLRK1	SAA1	XCL2
CARD16	CTSW	HLA-DOB	LAG3	SAMD3	ZBP1
CARD17	CXCL1	HLA-E	LAMP3	SAMD9L	ZFP57
CASP1	CXCL10	HLA-F	LILRA5	SCML4	CADM2
CASP5	CXCL11	IDO1	MX1	SIGLEC1	COL2A1
CCL2	CXCR3	IDO2	MZB1	SIGLEC14	PRKAA2
CCL4	CXCR6	IFI27	NCF1	SIRPB1	RIMS4
CCL5	DPT	IFI30	NCR1	SIRPG	SNAP25

PNRC					
ALOX5	CD69	EOMES	IL2RA	P2RY10	SLFN12L
ANKRD22	CD7	ETV7	IL2RG	P2RY6	SNX20
APBB1IP	CD70	EVI2B	ITGAL	PARP15	SP140
APOBEC3H	CD74	FFAR4	ITGB2	PRDM8	SPIB
APOL1	CD80	FYB1	ITK	PTPN22	TGFBI
ARHGAP9	CD8B	GBP5	JCHAIN	PTPN7	TMEM150B
BATF	CD96	GPR141	KDELR3	PYHIN1	TNFRSF9
BCL2A1	CDCP1	GPR171	LAIR2	RAC2	TNFSF14
C1R	CFB	GPR18	LAPTM5	RGS1	TRAF3IP3
C1S	CHI3L2	GPR84	LAX1	RHOH	TRAT1
C2	CLEC12A	HK3	LCK	RNASE2	TREM2
CASP5	CLECL1	HLA-DOA	LIPM	SAA2	TYMP
CCL5	CRTAM	HLA-DOB	LTB	SECTM1	UBASH3A
CCR4	CSF3R	HLA-DQA2	LY9	SH2D1A	UBD
CCR5	CST7	HLA-DQB2	LYZ	SH2D2A	VAV1
CD2	CSTA	HSH2D	METTL7B	SIGLEC1	XCR1
CD300LF	CTLA4	IBSP	MILR1	SIRPG	ZBP1
CD37	CTSW	ICOS	MZB1	SIT1	ZNF683
CD3D	CXCL10	IFI30	NCF1	SLAMF1	CALY
CD3E	CXCL11	IGLL5	NCF4	SLAMF6	JAKMIP1

CD3G	CXCL9	IKZF3	OASL	SLAMF7	PACSIN1
CD48	CXCR3	IL12RB1	OR2I1P	SLAMF8	SGSM1
CD52	CXCR6	IL21R	OSCAR	SLC52A1	SLCO5A1
CD53					

Table S3: GO enrichment analysis of PPRC hub genes

ID	Description	pvalue	Count
GO:0034341	response to interferon-gamma	7.69E-24	27
GO:0071346	cellular response to interferon-gamma	8.79E-24	26
GO:0042110	T cell activation	1.39E-21	35
GO:0009615	response to virus	5.01E-19	29
GO:0060337	type I interferon signaling pathway	7.86E-19	18
GO:0071357	cellular response to type I interferon	9.59E-19	18
GO:0034340	response to type I interferon	2.51E-18	18
GO:0002831	regulation of response to biotic stimulus	3.69E-18	30
GO:0002683	negative regulation of immune system process	1.16E-17	29
GO:0045088	regulation of innate immune response	2.30E-17	26
GO:0051607	defense response to virus	3.31E-17	24
GO:0140546	defense response to symbiont	3.31E-17	24
GO:0031341	regulation of cell killing	3.68E-17	17
GO:0002449	lymphocyte mediated immunity	6.04E-17	27
GO:0001906	cell killing	4.06E-16	20
GO:0002715	regulation of natural killer cell mediated immunity	4.06E-16	13
GO:0001909	leukocyte mediated cytotoxicity	4.51E-16	17
GO:0002697	regulation of immune effector process	5.24E-16	29
GO:0031343	positive regulation of cell killing	6.12E-16	14
GO:0001910	regulation of leukocyte mediated cytotoxicity	6.76E-16	15
GO:0007159	leukocyte cell-cell adhesion	8.88E-16	26
GO:0002228	natural killer cell mediated immunity	1.55E-15	14
GO:0042269	regulation of natural killer cell mediated cytotoxicity	5.20E-15	12
GO:0042267	natural killer cell mediated cytotoxicity	2.43E-14	13
GO:0031349	positive regulation of defense response	5.36E-14	24
GO:0050863	regulation of T cell activation	5.70E-14	23
GO:0001912	positive regulation of leukocyte mediated cytotoxicity	9.89E-14	12
GO:0002703	regulation of leukocyte mediated immunity	1.03E-13	19
GO:0002706	regulation of lymphocyte mediated immunity	1.22E-13	17
GO:0060333	interferon-gamma-mediated signaling pathway	1.39E-13	14
GO:0072676	lymphocyte migration	3.31E-13	15
GO:1903037	regulation of leukocyte cell-cell adhesion	6.16E-13	22
GO:0002705	positive regulation of leukocyte mediated immunity	7.83E-13	15
GO:0019079	viral genome replication	1.25E-12	15
GO:0002708	positive regulation of lymphocyte mediated immunity	1.59E-12	14
GO:0019058	viral life cycle	1.78E-12	22
GO:0045071	negative regulation of viral genome replication	2.45E-12	11
GO:0002699	positive regulation of immune effector process	2.66E-12	18
GO:0001819	positive regulation of cytokine production	3.30E-12	24
GO:0002716	negative regulation of natural killer cell mediated imm	3.55E-12	8
GO:0045089	positive regulation of innate immune response	3.62E-12	18
GO:0002833	positive regulation of response to biotic stimulus	4.49E-12	19
GO:0002460	adaptive immune response based on somatic recombin	5.11E-12	22
GO:0050867	positive regulation of cell activation	6.95E-12	23
GO:0070663	regulation of leukocyte proliferation	1.34E-11	18
GO:0002456	T cell mediated immunity	1.64E-11	13
GO:0048247	lymphocyte chemotaxis	1.76E-11	11
GO:0002696	positive regulation of leukocyte activation	2.89E-11	22
GO:0071674	mononuclear cell migration	3.19E-11	16
GO:0070098	chemokine-mediated signaling pathway	3.54E-11	12
GO:0048525	negative regulation of viral process	4.06E-11	12
GO:0002704	negative regulation of leukocyte mediated immunity	5.64E-11	10
GO:0002819	regulation of adaptive immune response	6.95E-11	15

GO:0002237	response to molecule of bacterial origin	9.69E-11	20
GO:1990868	response to chemokine	1.14E-10	12
GO:1990869	cellular response to chemokine	1.14E-10	12
GO:1903900	regulation of viral life cycle	1.21E-10	14
GO:0022407	regulation of cell-cell adhesion	1.51E-10	22
GO:0050870	positive regulation of T cell activation	1.65E-10	16
GO:0045953	negative regulation of natural killer cell mediated cytot	1.66E-10	7
GO:0030217	T cell differentiation	1.72E-10	17
GO:0030593	neutrophil chemotaxis	2.34E-10	12
GO:0002707	negative regulation of lymphocyte mediated immunity	3.05E-10	9
GO:0050670	regulation of lymphocyte proliferation	3.06E-10	16
GO:0045069	regulation of viral genome replication	3.27E-10	11
GO:0002717	positive regulation of natural killer cell mediated immu	3.37E-10	8
GO:0032944	regulation of mononuclear cell proliferation	3.49E-10	16
GO:0030595	leukocyte chemotaxis	4.25E-10	16
GO:0001913	T cell mediated cytotoxicity	5.63E-10	9
GO:1903039	positive regulation of leukocyte cell-cell adhesion	7.09E-10	16
GO:0001911	negative regulation of leukocyte mediated cytotoxicity	8.62E-10	7
GO:0070661	leukocyte proliferation	9.14E-10	18
GO:0051251	positive regulation of lymphocyte activation	1.12E-09	19
GO:0032496	response to lipopolysaccharide	1.84E-09	18
GO:1990266	neutrophil migration	1.87E-09	12
GO:0001914	regulation of T cell mediated cytotoxicity	1.97E-09	8
GO:0071621	granulocyte chemotaxis	2.05E-09	12
GO:0050792	regulation of viral process	2.48E-09	14
GO:0002822	regulation of adaptive immune response based on soma	2.52E-09	13
GO:0046634	regulation of alpha-beta T cell activation	2.78E-09	11
GO:0002709	regulation of T cell mediated immunity	2.98E-09	10
GO:0031342	negative regulation of cell killing	3.22E-09	7
GO:0045954	positive regulation of natural killer cell mediated cytot	3.22E-09	7
GO:1901623	regulation of lymphocyte chemotaxis	3.22E-09	7
GO:0042590	antigen processing and presentation of exogenous pept	3.83E-09	10
GO:0060326	cell chemotaxis	4.84E-09	17
GO:0043903	regulation of biological process involved in symbiotic	5.23E-09	14
GO:0042129	regulation of T cell proliferation	6.74E-09	13
GO:0019885	antigen processing and presentation of endogenous pep	7.13E-09	6
GO:0022409	positive regulation of cell-cell adhesion	7.68E-09	16
GO:0030098	lymphocyte differentiation	7.99E-09	18
GO:0045824	negative regulation of innate immune response	9.37E-09	9
GO:0046651	lymphocyte proliferation	1.04E-08	16
GO:1903131	mononuclear cell differentiation	1.17E-08	19
GO:0032943	mononuclear cell proliferation	1.21E-08	16
GO:0002483	antigen processing and presentation of endogenous pep	1.54E-08	6
GO:2001185	regulation of CD8-positive, alpha-beta T cell activation	1.54E-08	6
GO:0097530	granulocyte migration	1.55E-08	12
GO:0046631	alpha-beta T cell activation	1.67E-08	12
GO:0006959	humoral immune response	2.01E-08	18
GO:0035747	natural killer cell chemotaxis	3.04E-08	5
GO:0002474	antigen processing and presentation of peptide antigen	3.11E-08	10
GO:0002824	positive regulation of adaptive immune response based	3.11E-08	10
GO:0002479	antigen processing and presentation of exogenous pept	3.40E-08	9
GO:0002832	negative regulation of response to biotic stimulus	3.43E-08	10
GO:0042098	T cell proliferation	4.02E-08	13
GO:0071222	cellular response to lipopolysaccharide	4.53E-08	13
GO:0002821	positive regulation of adaptive immune response	5.00E-08	10
GO:0070555	response to interleukin-1	7.67E-08	13

GO:0071219	cellular response to molecule of bacterial origin	9.08E-08	13
GO:0002718	regulation of cytokine production involved in immune	9.26E-08	9
GO:0045785	positive regulation of cell adhesion	1.09E-07	18
GO:0002478	antigen processing and presentation of exogenous pept	1.13E-07	12
GO:0019883	antigen processing and presentation of endogenous ant	1.24E-07	6
GO:0071356	cellular response to tumor necrosis factor	1.35E-07	15
GO:0071347	cellular response to interleukin-1	1.36E-07	12
GO:0002429	immune response-activating cell surface receptor signa	1.41E-07	19
GO:0002757	immune response-activating signal transduction	1.41E-07	19
GO:0097529	myeloid leukocyte migration	1.48E-07	13
GO:0070665	positive regulation of leukocyte proliferation	1.53E-07	11
GO:0036037	CD8-positive, alpha-beta T cell activation	1.58E-07	6
GO:0050777	negative regulation of immune response	1.75E-07	11
GO:0019884	antigen processing and presentation of exogenous anti[illegible]	1.83E-07	12
GO:0072678	T cell migration	1.85E-07	8
GO:0032102	negative regulation of response to external stimulus	1.93E-07	17
GO:0050766	positive regulation of phagocytosis	2.09E-07	8
GO:0002367	cytokine production involved in immune response	2.25E-07	9
GO:0001916	positive regulation of T cell mediated cytotoxicity	2.49E-07	6
GO:0002698	negative regulation of immune effector process	2.87E-07	10
GO:0048002	antigen processing and presentation of peptide antigen	3.06E-07	12
GO:0071216	cellular response to biotic stimulus	3.20E-07	13
GO:0019882	antigen processing and presentation	3.36E-07	13
GO:0034612	response to tumor necrosis factor	3.67E-07	15
GO:0031348	negative regulation of defense response	3.70E-07	13
GO:0050727	regulation of inflammatory response	3.78E-07	16
GO:0042102	positive regulation of T cell proliferation	3.86E-07	9
GO:0001818	negative regulation of cytokine production	3.92E-07	16
GO:0050852	T cell receptor signaling pathway	5.25E-07	12
GO:0002711	positive regulation of T cell mediated immunity	5.33E-07	7
GO:0050671	positive regulation of lymphocyte proliferation	5.88E-07	10
GO:0032946	positive regulation of mononuclear cell proliferation	6.30E-07	10
GO:0046640	regulation of alpha-beta T cell proliferation	6.78E-07	6
GO:0006968	cellular defense response	6.95E-07	7
GO:0048245	eosinophil chemotaxis	7.19E-07	5
GO:0032649	regulation of interferon-gamma production	7.50E-07	9
GO:0043368	positive T cell selection	9.67E-07	6
GO:0031295	T cell costimulation	1.01E-06	7
GO:0032609	interferon-gamma production	1.10E-06	9
GO:0046633	alpha-beta T cell proliferation	1.15E-06	6
GO:0002700	regulation of production of molecular mediator of imm	1.21E-06	10
GO:0002695	negative regulation of leukocyte activation	1.21E-06	11
GO:0031294	lymphocyte costimulation	1.29E-06	7
GO:0070383	DNA cytosine deamination	1.51E-06	4
GO:0050868	negative regulation of T cell activation	1.84E-06	9
GO:0051250	negative regulation of lymphocyte activation	1.85E-06	10
GO:0046135	pyrimidine nucleoside catabolic process	2.02E-06	5
GO:0072677	eosinophil migration	2.02E-06	5
GO:2000401	regulation of lymphocyte migration	2.02E-06	7
GO:0050851	antigen receptor-mediated signaling pathway	2.30E-06	14
GO:0052548	regulation of endopeptidase activity	2.76E-06	16
GO:0050764	regulation of phagocytosis	2.87E-06	8
GO:0050866	negative regulation of cell activation	3.05E-06	11
GO:0002548	monocyte chemotaxis	3.41E-06	7
GO:0006216	cytidine catabolic process	3.50E-06	4
GO:0009972	cytidine deamination	3.50E-06	4

GO:0016554	cytidine to uridine editing	3.50E-06	4
GO:0046087	cytidine metabolic process	3.50E-06	4
GO:0002702	positive regulation of production of molecular mediato	4.25E-06	8
GO:0070227	lymphocyte apoptotic process	4.56E-06	7
GO:0010818	T cell chemotaxis	4.70E-06	5
GO:0070234	positive regulation of T cell apoptotic process	5.02E-06	4
GO:0045058	T cell selection	5.55E-06	6
GO:0002507	tolerance induction	5.67E-06	5
GO:0045619	regulation of lymphocyte differentiation	6.18E-06	10
GO:1903038	negative regulation of leukocyte cell-cell adhesion	6.25E-06	9
GO:0070231	T cell apoptotic process	6.28E-06	6
GO:0052547	regulation of peptidase activity	6.37E-06	16
GO:0045006	DNA deamination	6.97E-06	4
GO:0046131	pyrimidine ribonucleoside metabolic process	6.97E-06	4
GO:0046133	pyrimidine ribonucleoside catabolic process	6.97E-06	4
GO:1903706	regulation of hemopoiesis	9.06E-06	15
GO:0019835	cytolysis	9.59E-06	5
GO:0045580	regulation of T cell differentiation	9.88E-06	9
GO:0002720	positive regulation of cytokine production involved in	9.99E-06	6
GO:0070228	regulation of lymphocyte apoptotic process	1.12E-05	6
GO:0045869	negative regulation of single stranded viral RNA replic	1.25E-05	4
GO:0070230	positive regulation of lymphocyte apoptotic process	1.25E-05	4
GO:0002724	regulation of T cell cytokine production	1.32E-05	5
GO:1902105	regulation of leukocyte differentiation	1.34E-05	12
GO:0009164	nucleoside catabolic process	1.53E-05	5
GO:0070232	regulation of T cell apoptotic process	1.53E-05	5
GO:0002688	regulation of leukocyte chemotaxis	1.66E-05	8
GO:0002369	T cell cytokine production	2.05E-05	5
GO:0006213	pyrimidine nucleoside metabolic process	2.05E-05	5
GO:0032615	interleukin-12 production	2.06E-05	6
GO:0032655	regulation of interleukin-12 production	2.06E-05	6
GO:0002643	regulation of tolerance induction	2.07E-05	4
GO:0070269	pyroptosis	2.07E-05	4
GO:2000403	positive regulation of lymphocyte migration	2.35E-05	5
GO:0002685	regulation of leukocyte migration	2.46E-05	10
GO:0032613	interleukin-10 production	2.49E-05	6
GO:0032612	interleukin-1 production	2.51E-05	8
GO:0002544	chronic inflammatory response	2.60E-05	4
GO:0045091	regulation of single stranded viral RNA replication via	2.60E-05	4
GO:0016553	base conversion or substitution editing	3.23E-05	4
GO:0039692	single stranded viral RNA replication via double stranc	3.23E-05	4
GO:0140131	positive regulation of lymphocyte chemotaxis	3.23E-05	4
GO:0072529	pyrimidine-containing compound catabolic process	3.47E-05	5
GO:0046635	positive regulation of alpha-beta T cell activation	3.57E-05	6
GO:0050729	positive regulation of inflammatory response	3.71E-05	8
GO:2000514	regulation of CD4-positive, alpha-beta T cell activatior	3.90E-05	6
GO:0035710	CD4-positive, alpha-beta T cell activation	4.37E-05	7
GO:0002710	negative regulation of T cell mediated immunity	4.80E-05	4
GO:0072503	cellular divalent inorganic cation homeostasis	4.91E-05	15
GO:0071887	leukocyte apoptotic process	4.97E-05	7
GO:1901658	glycosyl compound catabolic process	5.55E-05	5
GO:0019730	antimicrobial humoral response	5.91E-05	8
GO:2000116	regulation of cysteine-type endopeptidase activity	6.51E-05	10
GO:0002418	immune response to tumor cell	6.88E-05	4
GO:0042454	ribonucleoside catabolic process	6.88E-05	4
GO:0032611	interleukin-1 beta production	7.15E-05	7

GO:0022408	negative regulation of cell-cell adhesion	7.58E-05	9
GO:0072507	divalent inorganic cation homeostasis	7.59E-05	15
GO:0042119	neutrophil activation	7.76E-05	15
GO:0002218	activation of innate immune response	7.92E-05	8
GO:0002440	production of molecular mediator of immune response	8.48E-05	11
GO:0071675	regulation of mononuclear cell migration	8.51E-05	7
GO:0002719	negative regulation of cytokine production involved in	9.53E-05	4
GO:0010528	regulation of transposition	9.53E-05	4
GO:0010529	negative regulation of transposition	9.53E-05	4
GO:2000108	positive regulation of leukocyte apoptotic process	9.53E-05	4
GO:0034656	nucleobase-containing small molecule catabolic proces	0.000103506	5
GO:2000106	regulation of leukocyte apoptotic process	0.000106086	6
GO:0007162	negative regulation of cell adhesion	0.000111504	11
GO:0002645	positive regulation of tolerance induction	0.000126831	3
GO:0098883	synapse pruning	0.000126831	3
GO:0080111	DNA demethylation	0.00012869	4
GO:0032652	regulation of interleukin-1 production	0.000131808	7
GO:0030101	natural killer cell activation	0.000139133	6
GO:0035821	modulation of process of other organism	0.000161964	7
GO:0002664	regulation of T cell tolerance induction	0.000167941	3
GO:0070664	negative regulation of leukocyte proliferation	0.000179852	6
GO:0002347	response to tumor cell	0.000193471	4
GO:0032196	transposition	0.000193471	4
GO:0035456	response to interferon-beta	0.000193471	4
GO:0043312	neutrophil degranulation	0.000201277	14
GO:0002283	neutrophil activation involved in immune response	0.000214402	14
GO:0035723	interleukin-15-mediated signaling pathway	0.000216819	3
GO:0071350	cellular response to interleukin-15	0.000216819	3
GO:0032663	regulation of interleukin-2 production	0.000228482	5
GO:0032653	regulation of interleukin-10 production	0.000247378	5
GO:0045589	regulation of regulatory T cell differentiation	0.000247955	4
GO:0006909	phagocytosis	0.000263624	12
GO:0032623	interleukin-2 production	0.000267437	5
GO:0002446	neutrophil mediated immunity	0.000269047	14
GO:0001959	regulation of cytokine-mediated signaling pathway	0.000271973	8
GO:0002475	antigen processing and presentation via MHC class Ib	0.00027405	3
GO:0002517	T cell tolerance induction	0.00027405	3
GO:0045059	positive thymic T cell selection	0.00027405	3
GO:0070672	response to interleukin-15	0.00027405	3
GO:0035510	DNA dealkylation	0.00027897	4
GO:0006874	cellular calcium ion homeostasis	0.000284595	13
GO:0071677	positive regulation of mononuclear cell migration	0.000311239	5
GO:0039694	viral RNA genome replication	0.000312674	4
GO:0032732	positive regulation of interleukin-1 production	0.000335076	5
GO:0045066	regulatory T cell differentiation	0.000349198	4
GO:0032729	positive regulation of interferon-gamma production	0.00036027	5
GO:0055074	calcium ion homeostasis	0.000367571	13
GO:0042130	negative regulation of T cell proliferation	0.00038687	5
GO:0010819	regulation of T cell chemotaxis	0.000415829	3
GO:0060759	regulation of response to cytokine stimulus	0.000422395	8
GO:0032651	regulation of interleukin-1 beta production	0.000422678	6
GO:0048246	macrophage chemotaxis	0.000431244	4
GO:0002701	negative regulation of production of molecular mediato	0.000477035	4
GO:0046632	alpha-beta T cell differentiation	0.000518035	6
GO:1901136	carbohydrate derivative catabolic process	0.000574746	8
GO:0002286	T cell activation involved in immune response	0.00062935	6

GO:0014002	astrocyte development	0.000635116	4
GO:0046006	regulation of activated T cell proliferation	0.000695171	4
GO:0002689	negative regulation of leukocyte chemotaxis	0.000704787	3
GO:0007204	positive regulation of cytosolic calcium ion concentrat	0.000714054	10
GO:0030449	regulation of complement activation	0.000758379	6
GO:0002221	pattern recognition receptor signaling pathway	0.000793257	8
GO:0002726	positive regulation of T cell cytokine production	0.000823462	3
GO:0009074	aromatic amino acid family catabolic process	0.000823462	3
GO:0070233	negative regulation of T cell apoptotic process	0.000823462	3
GO:0150146	cell junction disassembly	0.000823462	3
GO:0061844	antimicrobial humoral immune response mediated by a	0.000834869	5
GO:0002220	innate immune response activating cell surface recepto	0.000867882	6
GO:0051928	positive regulation of calcium ion transport	0.000867882	6
GO:0050798	activated T cell proliferation	0.000899353	4
GO:0002758	innate immune response-activating signal transduction	0.000906965	6
GO:0035455	response to interferon-alpha	0.000954107	3
GO:0046641	positive regulation of alpha-beta T cell proliferation	0.000954107	3
GO:0001774	microglial cell activation	0.000975883	4
GO:0002823	negative regulation of adaptive immune response basec	0.000975883	4
GO:0043367	CD4-positive, alpha-beta T cell differentiation	0.000990629	5
GO:0050672	negative regulation of lymphocyte proliferation	0.000990629	5
GO:0032945	negative regulation of mononuclear cell proliferation	0.001047052	5
GO:0032069	regulation of nuclease activity	0.00109717	3
GO:0045061	thymic T cell selection	0.00109717	3
GO:0050860	negative regulation of T cell receptor signaling pathwa	0.00109717	3
GO:0072527	pyrimidine-containing compound metabolic process	0.001230662	5
GO:0032693	negative regulation of interleukin-10 production	0.001253085	3
GO:0050920	regulation of chemotaxis	0.001277591	8
GO:0043370	regulation of CD4-positive, alpha-beta T cell differenti	0.001328054	4
GO:0097300	programmed necrotic cell death	0.001328054	4
GO:0019724	B cell mediated immunity	0.00135184	8

Table S4: GO enrichment analysis of PNRC hub genes

ID	Description	pvalue	Count
GO:0042110	T cell activation	4.65E-26	35
GO:0050863	regulation of T cell activation	7.05E-18	24
GO:0030217	T cell differentiation	4.53E-17	21
GO:1903131	mononuclear cell differentiation	1.16E-16	25
GO:0030098	lymphocyte differentiation	6.76E-16	23
GO:0007159	leukocyte cell-cell adhesion	1.09E-15	23
GO:1903037	regulation of leukocyte cell-cell adhesion	1.68E-14	21
GO:1903039	positive regulation of leukocyte cell-cell adhesion	5.64E-14	18
GO:0022409	positive regulation of cell-cell adhesion	9.57E-13	18
GO:0050870	positive regulation of T cell activation	2.07E-12	16
GO:0022407	regulation of cell-cell adhesion	3.87E-12	21
GO:0002696	positive regulation of leukocyte activation	6.75E-12	20
GO:0050867	positive regulation of cell activation	1.10E-11	20
GO:0045785	positive regulation of cell adhesion	1.93E-11	20
GO:0002697	regulation of immune effector process	9.62E-11	20
GO:0043368	positive T cell selection	1.27E-10	8
GO:0050852	T cell receptor signaling pathway	1.90E-10	14
GO:0060326	cell chemotaxis	5.05E-10	16
GO:0050670	regulation of lymphocyte proliferation	5.45E-10	14
GO:0051251	positive regulation of lymphocyte activation	5.65E-10	17
GO:0070098	chemokine-mediated signaling pathway	6.11E-10	10
GO:0032944	regulation of mononuclear cell proliferation	6.13E-10	14
GO:0070661	leukocyte proliferation	6.71E-10	16
GO:0050851	antigen receptor-mediated signaling pathway	1.11E-09	16
GO:1990266	neutrophil migration	1.11E-09	11
GO:0046651	lymphocyte proliferation	1.44E-09	15
GO:0045058	T cell selection	1.47E-09	8
GO:1990868	response to chemokine	1.61E-09	10
GO:1990869	cellular response to chemokine	1.61E-09	10
GO:0032943	mononuclear cell proliferation	1.66E-09	15
GO:0070663	regulation of leukocyte proliferation	1.69E-09	14
GO:0002683	negative regulation of immune system process	3.69E-09	17
GO:0045619	regulation of lymphocyte differentiation	4.04E-09	12
GO:0030595	leukocyte chemotaxis	7.47E-09	13
GO:0097530	granulocyte migration	7.94E-09	11
GO:0046631	alpha-beta T cell activation	8.52E-09	11
GO:1902105	regulation of leukocyte differentiation	1.10E-08	14
GO:0042098	T cell proliferation	1.37E-08	12
GO:0042129	regulation of T cell proliferation	2.98E-08	11
GO:1903706	regulation of hemopoiesis	3.86E-08	16
GO:0007204	positive regulation of cytosolic calcium ion concentratior	4.14E-08	14
GO:0030593	neutrophil chemotaxis	4.70E-08	9
GO:0097529	myeloid leukocyte migration	4.72E-08	12
GO:0002699	positive regulation of immune effector process	4.96E-08	12
GO:0002429	immune response-activating cell surface receptor signalir	4.99E-08	17
GO:0002757	immune response-activating signal transduction	4.99E-08	17
GO:0002695	negative regulation of leukocyte activation	6.78E-08	11
GO:0006909	phagocytosis	8.06E-08	15
GO:0045580	regulation of T cell differentiation	8.49E-08	10
GO:0002286	T cell activation involved in immune response	9.03E-08	9
GO:0031295	T cell costimulation	1.46E-07	7
GO:0051480	regulation of cytosolic calcium ion concentration	1.50E-07	14
GO:0050866	negative regulation of cell activation	1.77E-07	11

GO:0045730	respiratory burst	1.80E-07	6
GO:0031294	lymphocyte costimulation	1.86E-07	7
GO:0071621	granulocyte chemotaxis	2.35E-07	9
GO:0002449	lymphocyte mediated immunity	2.60E-07	14
GO:0050856	regulation of T cell receptor signaling pathway	3.46E-07	6
GO:0001819	positive regulation of cytokine production	4.69E-07	15
GO:0050671	positive regulation of lymphocyte proliferation	4.85E-07	9
GO:0042102	positive regulation of T cell proliferation	4.94E-07	8
GO:0032946	positive regulation of mononuclear cell proliferation	5.16E-07	9
GO:0006874	cellular calcium ion homeostasis	5.41E-07	15
GO:0055074	calcium ion homeostasis	7.58E-07	15
GO:0032649	regulation of interferon-gamma production	8.97E-07	8
GO:0046632	alpha-beta T cell differentiation	8.97E-07	8
GO:0002285	lymphocyte activation involved in immune response	9.34E-07	10
GO:0070665	positive regulation of leukocyte proliferation	1.05E-06	9
GO:0032609	interferon-gamma production	1.27E-06	8
GO:0072503	cellular divalent inorganic cation homeostasis	1.52E-06	15
GO:0002831	regulation of response to biotic stimulus	1.64E-06	14
GO:0050868	negative regulation of T cell activation	2.01E-06	8
GO:0006968	cellular defense response	2.15E-06	6
GO:0045059	positive thymic T cell selection	2.24E-06	4
GO:0072540	T-helper 17 cell lineage commitment	2.24E-06	4
GO:0045088	regulation of innate immune response	2.41E-06	12
GO:0072507	divalent inorganic cation homeostasis	2.45E-06	15
GO:0006959	humoral immune response	2.96E-06	13
GO:0002295	T-helper cell lineage commitment	5.24E-06	4
GO:1903038	negative regulation of leukocyte cell-cell adhesion	6.08E-06	8
GO:0032729	positive regulation of interferon-gamma production	6.44E-06	6
GO:0050854	regulation of antigen receptor-mediated signaling pathwa	7.05E-06	6
GO:0002228	natural killer cell mediated immunity	7.70E-06	6
GO:0002287	alpha-beta T cell activation involved in immune response	7.70E-06	6
GO:0002293	alpha-beta T cell differentiation involved in immune resp	7.70E-06	6
GO:0071674	mononuclear cell migration	8.21E-06	9
GO:0002495	antigen processing and presentation of peptide antigen vi	8.35E-06	7
GO:0002504	antigen processing and presentation of peptide or polysac	8.90E-06	7
GO:0048002	antigen processing and presentation of peptide antigen	9.71E-06	9
GO:0002577	regulation of antigen processing and presentation	1.05E-05	4
GO:0043373	CD4-positive, alpha-beta T cell lineage commitment	1.05E-05	4
GO:0034341	response to interferon-gamma	1.10E-05	9
GO:0002292	T cell differentiation involved in immune response	1.27E-05	6
GO:0002363	alpha-beta T cell lineage commitment	1.29E-05	4
GO:0051250	negative regulation of lymphocyte activation	1.30E-05	8
GO:0051209	release of sequestered calcium ion into cytosol	1.45E-05	7
GO:0051283	negative regulation of sequestering of calcium ion	1.54E-05	7
GO:0043369	CD4-positive or CD8-positive, alpha-beta T cell lineage c	1.57E-05	4
GO:0045061	thymic T cell selection	1.57E-05	4
GO:0050860	negative regulation of T cell receptor signaling pathway	1.57E-05	4
GO:0051282	regulation of sequestering of calcium ion	1.72E-05	7
GO:0051208	sequestering of calcium ion	2.15E-05	7
GO:0002715	regulation of natural killer cell mediated immunity	2.17E-05	5
GO:0001961	positive regulation of cytokine-mediated signaling pathw	2.40E-05	5
GO:0030101	natural killer cell activation	2.84E-05	6
GO:0043277	apoptotic cell clearance	2.93E-05	5
GO:0045954	positive regulation of natural killer cell mediated cytotox	3.13E-05	4
GO:0060142	regulation of syncytium formation by plasma membrane f	3.13E-05	4
GO:0002360	T cell lineage commitment	3.66E-05	4

GO:0010818	T cell chemotaxis	3.66E-05	4
GO:0002478	antigen processing and presentation of exogenous peptide	3.71E-05	8
GO:0001906	cell killing	4.02E-05	8
GO:0001912	positive regulation of leukocyte mediated cytotoxicity	4.24E-05	5
GO:0002507	tolerance induction	4.24E-05	4
GO:0019882	antigen processing and presentation	4.28E-05	9
GO:0097553	calcium ion transmembrane import into cytosol	4.62E-05	7
GO:0060760	positive regulation of response to cytokine stimulus	4.63E-05	5
GO:0001773	myeloid dendritic cell activation	4.89E-05	4
GO:0036336	dendritic cell migration	4.89E-05	4
GO:0072539	T-helper 17 cell differentiation	4.89E-05	4
GO:0019884	antigen processing and presentation of exogenous antigen	5.08E-05	8
GO:0050858	negative regulation of antigen receptor-mediated signalin	5.62E-05	4
GO:0022408	negative regulation of cell-cell adhesion	5.91E-05	8
GO:0002460	adaptive immune response based on somatic recombinati	5.91E-05	11
GO:0002717	positive regulation of natural killer cell mediated immuni	6.41E-05	4
GO:0019886	antigen processing and presentation of exogenous peptide	7.19E-05	6
GO:0046634	regulation of alpha-beta T cell activation	8.04E-05	6
GO:0019722	calcium-mediated signaling	8.18E-05	8
GO:0031343	positive regulation of cell killing	8.18E-05	5
GO:0072538	T-helper 17 type immune response	8.25E-05	4
GO:0042093	T-helper cell differentiation	8.83E-05	5
GO:0042267	natural killer cell mediated cytotoxicity	8.83E-05	5
GO:0051607	defense response to virus	9.63E-05	9
GO:0140546	defense response to symbiont	9.63E-05	9
GO:0002294	CD4-positive, alpha-beta T cell differentiation involved i	0.000102324	5
GO:0072678	T cell migration	0.000102324	5
GO:0060402	calcium ion transport into cytosol	0.0001024	7
GO:0002685	regulation of leukocyte migration	0.000104213	8
GO:0042113	B cell activation	0.000107014	10
GO:0050766	positive regulation of phagocytosis	0.000109956	5
GO:1902107	positive regulation of leukocyte differentiation	0.000111119	7
GO:1903708	positive regulation of hemopoiesis	0.000111119	7
GO:0002517	T cell tolerance induction	0.000117864	3
GO:0035589	G protein-coupled purinergic nucleotide receptor signalin	0.000117864	3
GO:0002703	regulation of leukocyte mediated immunity	0.000119132	8
GO:0051651	maintenance of location in cell	0.000119132	8
GO:0071219	cellular response to molecule of bacterial origin	0.000119132	8
GO:0001909	leukocyte mediated cytotoxicity	0.000149585	6
GO:0051281	positive regulation of release of sequestered calcium ion	0.000177344	4
GO:0060263	regulation of respiratory burst	0.000179465	3
GO:0019058	viral life cycle	0.000182085	10
GO:0045089	positive regulation of innate immune response	0.000185939	8
GO:0072676	lymphocyte migration	0.000189572	6
GO:1902622	regulation of neutrophil migration	0.000214847	4
GO:0042119	neutrophil activation	0.00022195	12
GO:0060401	cytosolic calcium ion transport	0.000228128	7
GO:0009615	response to virus	0.000233812	10
GO:0001910	regulation of leukocyte mediated cytotoxicity	0.000239849	5
GO:0007162	negative regulation of cell adhesion	0.000248151	9
GO:0071216	cellular response to biotic stimulus	0.000250443	8
GO:0042269	regulation of natural killer cell mediated cytotoxicity	0.000257748	4
GO:0002523	leukocyte migration involved in inflammatory response	0.000258821	3
GO:1901739	regulation of myoblast fusion	0.000258821	3
GO:0002705	positive regulation of leukocyte mediated immunity	0.000259238	6
GO:0071346	cellular response to interferon-gamma	0.000262301	7

GO:0043367	CD4-positive, alpha-beta T cell differentiation	0.000269623	5
GO:0043652	engulfment of apoptotic cell	0.000305768	3
GO:0019932	second-messenger-mediated signaling	0.000332612	9
GO:0045582	positive regulation of T cell differentiation	0.000396365	5
GO:0050864	regulation of B cell activation	0.000442513	7
GO:0050921	positive regulation of chemotaxis	0.000458037	6
GO:0046718	viral entry into host cell	0.000475815	6
GO:0002833	positive regulation of response to biotic stimulus	0.000494742	8
GO:0071222	cellular response to lipopolysaccharide	0.000500257	7
GO:0032496	response to lipopolysaccharide	0.000514084	9
GO:0010524	positive regulation of calcium ion transport into cytosol	0.000529412	4
GO:0050764	regulation of phagocytosis	0.00053698	5
GO:0060143	positive regulation of syncytium formation by plasma me	0.000547433	3
GO:0000768	syncytium formation by plasma membrane fusion	0.000568665	4
GO:0140253	cell-cell fusion	0.000568665	4
GO:0042100	B cell proliferation	0.000591286	5
GO:0002407	dendritic cell chemotaxis	0.000622423	3
GO:0031341	regulation of cell killing	0.000649614	5
GO:0006949	syncytium formation	0.000653302	4
GO:0043312	neutrophil degranulation	0.000653372	11
GO:0002283	neutrophil activation involved in immune response	0.000687368	11
GO:0002260	lymphocyte homeostasis	0.000698802	4
GO:0035710	CD4-positive, alpha-beta T cell activation	0.000712149	5
GO:0070374	positive regulation of ERK1 and ERK2 cascade	0.000730572	7
GO:0045621	positive regulation of lymphocyte differentiation	0.000745053	5
GO:0044409	entry into host	0.000759593	6
GO:0002237	response to molecule of bacterial origin	0.000785991	9
GO:0002446	neutrophil mediated immunity	0.00082491	11
GO:0045576	mast cell activation	0.000848723	4
GO:0002706	regulation of lymphocyte mediated immunity	0.000868988	6
GO:0036037	CD8-positive, alpha-beta T cell activation	0.000885714	3
GO:0090025	regulation of monocyte chemotaxis	0.000885714	3
GO:0030888	regulation of B cell proliferation	0.000903366	4
GO:0002708	positive regulation of lymphocyte mediated immunity	0.001008192	5
GO:0031349	positive regulation of defense response	0.001058913	9
GO:0050920	regulation of chemotaxis	0.001065175	7
GO:0048247	lymphocyte chemotaxis	0.001082122	4
GO:0019724	B cell mediated immunity	0.001121461	7
GO:0050727	regulation of inflammatory response	0.001165332	9
GO:0050850	positive regulation of calcium-mediated signaling	0.001210579	3
GO:0042130	negative regulation of T cell proliferation	0.001214232	4
GO:0046635	positive regulation of alpha-beta T cell activation	0.001214232	4

Table S5: Univariate Cox regression analysis of hub genes from PPRC

Symbol	HR	HR.95L	HR.95H	pvalue
ACSL5	0.832	0.783	0.883	1.86E-09
ADAMDEC1	0.913	0.882	0.945	2.53E-07
ANKRD22	0.858	0.823	0.894	3.62E-13
AOAH	0.921	0.873	0.972	0.002772863
APBB1IP	0.901	0.847	0.959	0.000965906
APOBEC3A	0.929	0.889	0.970	0.000946398
APOBEC3D	0.888	0.829	0.951	0.000634954
APOBEC3G	0.879	0.826	0.936	5.69E-05
APOL1	0.894	0.842	0.948	0.000180475
APOL3	0.813	0.769	0.859	1.48E-13
APOL6	0.777	0.731	0.826	6.12E-16
B2M	0.862	0.795	0.934	0.00029483
BCL2L14	0.773	0.737	0.812	3.62E-25
BIRC3	0.873	0.831	0.917	5.21E-08
BST2	0.885	0.841	0.932	3.39E-06
CALHM6	0.915	0.868	0.963	0.000720697
CARD16	1.090	1.025	1.159	0.006231625
CARD17	0.892	0.842	0.945	0.000100392
CCL2	1.063	1.003	1.127	0.039624461
CCL4	0.945	0.895	0.998	0.043560111
CCL5	0.908	0.866	0.952	5.68E-05
CCL8	0.935	0.893	0.978	0.003775598
CCR5	0.888	0.846	0.932	1.69E-06
CD2	0.882	0.845	0.921	9.98E-09
CD247	0.896	0.853	0.940	8.96E-06
CD274	0.870	0.819	0.925	8.14E-06
CD3D	0.905	0.867	0.944	5.05E-06
CD3E	0.910	0.872	0.950	1.72E-05
CD3G	0.891	0.855	0.928	3.84E-08
CD48	0.867	0.823	0.913	7.32E-08
CD80	0.854	0.808	0.901	1.09E-08
CD8A	0.896	0.857	0.937	1.25E-06
CD8B	0.903	0.866	0.942	2.14E-06
CD96	0.889	0.850	0.930	2.60E-07
CFB	0.912	0.882	0.943	7.15E-08
CHI3L1	0.941	0.908	0.975	0.000771821
CHIT1	0.919	0.888	0.950	9.10E-07
CLEC4D	0.947	0.899	0.997	0.039659287
CLEC4E	0.912	0.872	0.953	4.94E-05
CRTAM	0.911	0.866	0.957	0.000226983
CST7	0.918	0.872	0.966	0.001004914
CTSW	0.933	0.890	0.979	0.004282534
CXCL1	1.085	1.049	1.122	2.17E-06
CXCL10	0.887	0.855	0.921	3.99E-10
CXCL11	0.874	0.842	0.907	7.96E-13
CXCR3	0.916	0.878	0.956	5.88E-05
CXCR6	0.886	0.844	0.931	1.41E-06
DPT	0.912	0.879	0.946	9.68E-07
DTHD1	0.900	0.861	0.942	5.28E-06
EPSTI1	0.897	0.850	0.947	8.35E-05
ETV7	0.919	0.874	0.966	0.000955557
FASLG	0.915	0.876	0.955	6.23E-05
FCRL6	0.891	0.845	0.940	2.49E-05

GBP1	0.867	0.824	0.912	2.47E-08
GBP2	0.760	0.717	0.805	8.54E-21
GBP4	0.858	0.819	0.900	1.92E-10
GBP5	0.900	0.864	0.937	2.59E-07
GBP6	0.910	0.866	0.956	0.000163867
GIMAP2	0.821	0.770	0.876	2.07E-09
GPR171	0.863	0.826	0.901	2.80E-11
GPR18	0.875	0.829	0.923	1.02E-06
GPR31	0.834	0.787	0.883	7.00E-10
GZMA	0.905	0.865	0.947	1.69E-05
GZMH	0.941	0.899	0.984	0.007802328
GZMK	0.908	0.874	0.944	8.74E-07
HLA.B	0.907	0.850	0.968	0.003241779
HLA.DOB	0.838	0.797	0.881	4.45E-12
IDO1	0.903	0.870	0.938	1.03E-07
IDO2	0.886	0.839	0.936	1.54E-05
IFI27	0.935	0.895	0.978	0.003156644
IFI44	0.884	0.834	0.937	3.33E-05
IFI44L	0.873	0.836	0.911	4.23E-10
IFI6	0.948	0.902	0.997	0.038244517
IFNG	0.934	0.898	0.972	0.000682404
IKZF3	0.878	0.841	0.916	1.90E-09
IL15RA	0.892	0.833	0.955	0.000986666
IL2RB	0.922	0.876	0.970	0.001798584
IL2RG	0.885	0.843	0.930	1.16E-06
IL4I1	0.950	0.903	1.000	0.04849726
IRF1	0.813	0.761	0.869	9.45E-10
ISG15	0.924	0.880	0.971	0.001654973
ITGAL	0.864	0.821	0.909	1.54E-08
ITGB7	0.915	0.865	0.969	0.00233788
JAKMIP1	0.907	0.868	0.948	1.46E-05
KIR3DL2	0.907	0.851	0.966	0.002361123
KLHDC7B	0.869	0.833	0.905	2.58E-11
KLRB1	0.855	0.808	0.905	6.01E-08
KLRC1	0.880	0.837	0.924	3.33E-07
KLRD1	0.873	0.829	0.919	2.30E-07
KLRK1	0.906	0.863	0.952	7.86E-05
LAMP3	0.901	0.861	0.942	5.00E-06
MX1	0.936	0.890	0.985	0.01128393
MZB1	0.887	0.860	0.915	4.99E-14
NCF1	0.935	0.885	0.988	0.016662245
NLRC5	0.905	0.843	0.972	0.006027019
NUGGC	0.919	0.878	0.961	0.000225916
OAS2	0.881	0.835	0.931	5.30E-06
OASL	0.896	0.853	0.941	1.25E-05
PATL2	0.927	0.874	0.983	0.011698531
PDZK1IP1	0.977	0.956	0.999	0.042606666
PLAAT4	0.847	0.813	0.882	2.15E-15
PTGDR	0.856	0.807	0.908	2.12E-07
PYHIN1	0.921	0.882	0.962	0.000202579
RSAD2	0.880	0.841	0.920	1.58E-08
RUFY4	0.889	0.848	0.932	9.98E-07
SAA1	0.951	0.928	0.975	9.09E-05
SAMD3	0.871	0.830	0.914	1.66E-08
SAMD9L	0.849	0.804	0.897	5.77E-09
SIRPG	0.923	0.887	0.961	0.000105136

SLA2	0.894	0.851	0.940	1.03E-05
SMTNL1	0.858	0.811	0.909	1.58E-07
SP140	0.875	0.829	0.923	1.00E-06
STAP1	0.858	0.819	0.899	1.49E-10
TBX21	0.918	0.876	0.962	0.000377666
THEMIS	0.883	0.848	0.920	1.94E-09
TIFAB	0.922	0.884	0.962	0.000148134
TIGIT	0.904	0.869	0.941	8.37E-07
TMPRSS3	0.928	0.905	0.953	1.42E-08
TNFSF13B	0.920	0.870	0.974	0.004058841
TRIM22	0.839	0.789	0.892	2.35E-08
TTC22	0.896	0.868	0.924	3.54E-12
TYMP	0.930	0.870	0.994	0.03288686
UBASH3A	0.914	0.874	0.955	6.56E-05
VSIR	1.119	1.043	1.200	0.00168267
XAF1	0.880	0.835	0.927	1.86E-06
XCL2	0.923	0.882	0.967	0.00066608
ZBP1	0.887	0.849	0.926	4.97E-08
ZFP57	0.942	0.912	0.972	0.000221094
CADM2	0.901	0.870	0.934	1.01E-08
COL2A1	0.966	0.939	0.993	0.014107639
PRKAA2	0.902	0.878	0.926	1.76E-14
RIMS4	0.927	0.902	0.952	2.10E-08

Table S6: Univariate Cox regression analysis of hub genes from PNRC

Symbol	HR	HR.95L	HR.95H	pvalue
ALOX5	1.143	1.092	1.197	1.08E-08
ANKRD22	1.178	1.131	1.226	1.80E-15
APOL1	1.040	1.008	1.074	0.01545585
ARHGAP9	1.160	1.086	1.238	9.34E-06
BATF	1.155	1.110	1.203	1.68E-12
BCL2A1	1.222	1.165	1.282	2.40E-16
C1R	1.268	1.211	1.328	6.44E-24
C1S	1.186	1.138	1.236	7.77E-16
C2	1.113	1.067	1.162	8.48E-07
CASP5	1.241	1.174	1.313	3.39E-14
CCR5	1.043	1.006	1.082	0.022156583
CD300LF	1.139	1.082	1.199	6.53E-07
CD37	1.122	1.049	1.199	0.000758975
CD48	1.041	1.007	1.075	0.015945474
CD53	1.110	1.047	1.178	0.000513326
CD69	1.066	1.025	1.108	0.001300142
CD7	1.064	1.021	1.109	0.002999838
CD74	1.071	1.024	1.121	0.002702866
CD80	1.123	1.077	1.171	6.62E-08
CDCP1	1.116	1.079	1.154	1.95E-10
CFB	1.050	1.014	1.088	0.005754032
CHI3L2	1.213	1.176	1.250	1.58E-35
CLEC12A	1.058	1.025	1.093	0.000556309
CLECL1	1.055	1.004	1.109	0.033629467
CSF3R	1.160	1.090	1.236	3.54E-06
CSTA	1.192	1.142	1.243	4.43E-16
CTLA4	1.076	1.035	1.119	0.000241562
CXCL10	1.070	1.041	1.100	1.12E-06
CXCL11	1.080	1.047	1.114	1.31E-06
ETV7	1.068	1.029	1.109	0.000571251
EVI2B	1.063	1.008	1.121	0.024762359
FFAR4	1.117	1.061	1.176	2.82E-05
GBP5	1.047	1.016	1.079	0.003046894
GPR141	1.059	1.020	1.100	0.002572659
GPR84	1.338	1.267	1.413	8.48E-26
HK3	1.252	1.184	1.323	1.85E-15
HSH2D	1.089	1.055	1.124	1.53E-07
IBSP	1.208	1.177	1.240	1.03E-45
ICOS	1.034	1.002	1.066	0.035991168
IFI30	1.203	1.148	1.261	1.36E-14
IGLL5	1.024	1.003	1.045	0.021663775
IL2RA	1.117	1.082	1.152	6.32E-12
IL2RG	1.055	1.021	1.091	0.001583791
ITGB2	1.123	1.058	1.191	0.000138913
ITK	1.035	1.004	1.067	0.028856955
KDELR3	1.153	1.109	1.198	6.60E-13
LAIR2	1.055	1.009	1.102	0.018817784
LAPTM5	1.165	1.092	1.243	3.64E-06
LIPM	1.166	1.117	1.217	2.29E-12
LYZ	1.055	1.028	1.083	5.79E-05
METTL7B	1.191	1.143	1.241	6.56E-17
MILR1	1.240	1.159	1.327	4.25E-10
MZB1	1.044	1.014	1.075	0.003797538

NCF1	1.202	1.133	1.275	9.20E-10
NCF4	1.235	1.147	1.329	1.92E-08
OASL	1.205	1.153	1.259	9.39E-17
OSCAR	1.358	1.265	1.458	2.45E-17
P2RY6	1.204	1.142	1.269	6.39E-12
PARP15	1.112	1.063	1.164	4.70E-06
PTPN22	1.104	1.065	1.144	6.45E-08
PTPN7	1.140	1.092	1.191	2.34E-09
RAC2	1.129	1.078	1.181	1.97E-07
RGS1	1.086	1.041	1.134	0.000145571
RHOH	1.075	1.029	1.122	0.001082991
RNASE2	1.322	1.261	1.386	2.45E-31
SAA2	1.141	1.117	1.165	1.05E-34
SECTM1	1.138	1.088	1.189	1.34E-08
SH2D2A	1.085	1.046	1.126	1.59E-05
SIGLEC1	1.103	1.049	1.159	0.00011111
SLAMF1	1.037	1.005	1.070	0.024159475
SLAMF8	1.123	1.077	1.171	4.76E-08
SLC52A1	1.186	1.134	1.241	9.62E-14
SNX20	1.086	1.036	1.138	0.000635117
SP140	1.126	1.063	1.194	5.97E-05
SPIB	1.096	1.059	1.133	1.35E-07
TGFBI	1.057	1.027	1.088	0.000155983
TMEM150B	1.115	1.065	1.168	3.13E-06
TNFSF14	1.106	1.071	1.142	1.05E-09
TREM2	1.181	1.116	1.249	6.13E-09
TYMP	1.122	1.076	1.169	5.64E-08
VAV1	1.115	1.046	1.189	0.000849197
XCR1	0.971	0.943	0.999	0.040721427
ZBP1	1.144	1.096	1.195	9.71E-10
CALY	1.037	1.014	1.060	0.001557352
SGSM1	0.747	0.714	0.782	3.91E-36

Table S7: Multivariate regression analysis of OS-related hub genes from PPRC

Symbol	coef	HR	HR.95L	HR.95H	pvalue
BCL2L14	-0.121	0.886	0.826	0.950	0.000640642
CCL2	0.105	1.111	1.029	1.198	0.006738371
CHIT1	-0.039	0.962	0.929	0.995	0.025833952
GBP2	-0.254	0.776	0.706	0.852	1.20E-07
GBP6	0.071	1.074	1.017	1.133	0.01016108
HLA.DOB	-0.074	0.928	0.860	1.002	0.054825355
PDZK1IP1	0.039	1.040	1.010	1.071	0.008411504
PLAAT4	-0.090	0.914	0.859	0.972	0.004187562
VSIR	0.228	1.256	1.147	1.375	8.87E-07
ZFP57	-0.029	0.971	0.941	1.002	0.064980025
PRKAA2	-0.091	0.913	0.884	0.943	3.42E-08

Table S8: Multivariate regression analysis of OS-related hub genes from PNRC

Symbol	coef	HR	HR.95L	HR.95H	pvalue
ANKRD22	0.124	1.132	1.013	1.265	0.028266987
APOL1	-0.158	0.854	0.778	0.937	0.000900206
BCL2A1	-0.132	0.876	0.791	0.970	0.01063182
C1R	0.143	1.154	1.040	1.281	0.006860712
CD300LF	0.252	1.287	1.108	1.494	0.000972829
CD48	-0.162	0.851	0.754	0.959	0.008324089
CHI3L2	0.086	1.090	1.032	1.151	0.001958512
CLECL1	-0.157	0.855	0.779	0.938	0.000927107
CSF3R	-0.243	0.784	0.666	0.924	0.003587056
CXCL11	0.098	1.103	1.026	1.186	0.008248398
ETV7	-0.082	0.921	0.833	1.019	0.109444726
EVI2B	0.463	1.588	1.274	1.979	3.86E-05
GBP5	-0.105	0.900	0.800	1.012	0.078320793
GPR84	0.092	1.097	0.968	1.243	0.14901179
IBSP	0.070	1.072	1.028	1.118	0.001048773
IFI30	0.107	1.113	0.999	1.240	0.051949079
IL2RA	0.093	1.097	1.029	1.170	0.004822728
ITGB2	-0.180	0.835	0.665	1.049	0.122158876
KDELR3	0.115	1.121	1.027	1.224	0.01043019
LAPTM5	-0.696	0.498	0.371	0.670	3.92E-06
LIPM	-0.116	0.891	0.801	0.990	0.032133357
MILR1	0.278	1.321	1.111	1.571	0.001666044
NCF1	0.127	1.136	0.976	1.322	0.100379459
OASL	0.128	1.137	1.037	1.246	0.006053046
P2RY6	0.095	1.100	0.989	1.223	0.079432647
PTPN7	0.397	1.487	1.266	1.748	1.43E-06
RAC2	0.176	1.192	1.000	1.422	0.050483735
RHOH	-0.182	0.834	0.718	0.969	0.017555653
RNASE2	0.142	1.153	1.026	1.295	0.016787636
SAA2	0.044	1.045	1.006	1.085	0.023687041
SH2D2A	0.100	1.105	0.988	1.237	0.08043452
SLAMF1	-0.148	0.862	0.770	0.966	0.010844045
SLAMF8	0.106	1.111	0.979	1.261	0.102442397
SNX20	-0.232	0.793	0.630	0.997	0.04694057
SP140	0.152	1.164	0.980	1.383	0.082689085
TGFBI	-0.069	0.933	0.873	0.998	0.044021979
VAV1	-0.297	0.743	0.599	0.921	0.006762155
XCR1	-0.191	0.826	0.767	0.889	4.01E-07
ZBP1	0.099	1.104	0.992	1.229	0.070478088

Table S9: Cutoff values of PP- or PN-Score from 8 different cancers

Cancer types	Subtypes	Cutoff values	Number of patients	
			Low	High
BRCA	PPRC	0.875263567	722	337
MESO		4.537745731	38	46
SKCM		1.129789111	148	258
GBM	PNRC	5.858684255	89	78
KIRC		0.941287221	365	163
LGG		1.368258587	420	104
PAAD		1.960776927	53	121
UVM		0.411547864	15	64

Table S10: Top 3 mutated genes of each cancer type

PPRC subtype	Genes	Mutation	Wildtype	Number of patients	Mutation frequency
BRCA	TP53	257	511	768	33.46%
	PIK3CA	315	630	945	33.33%
	CDH1	104	664	768	13.54%
MESO	BAP1	18	60	78	23.08%
	NF2	19	59	78	24.36%
	TP53	14	64	78	17.95%
SKCM	BRAF	204	195	399	51.13%
	FAT4	154	245	399	38.60%
	GRIN2A	136	263	399	34.09%

PNRC subtype	Genes	Mutation	Wildtype	Number of patients	Mutation frequency
GBMLGG	IDH1	394	257	651	60.52%
	TP53	295	356	651	45.31%
	ATRX	203	448	651	31.18%
KIRC	VHL	167	193	360	46.39%
	PBRM1	149	211	360	41.39%
	SETD2	50	310	360	13.89%
PAAD	KRAS	108	57	165	65.45%
	TP53	101	64	165	61.21%
	SMAD4	36	129	165	21.82%
UVM	GNAQ	40	39	79	50.63%
	GNA11	35	44	79	44.30%
	BAP1	13	66	79	16.46%

Table S11: Correlation between π score and IC50 of 198 chemotherapy drugs

	BRCA		MESO		
	R	FDR	R	FDR	R
Camptothecin_1003	0.146	1.87E-06	0.379	0.0003825	0.336
Vinblastine_1004	0.192	2.72E-10	0.460	1.08E-05	0.285
Cisplatin_1005	0.149	1.17E-06	0.394	0.000212472	0.323
Cytarabine_1006	0.241	1.81E-15	0.421	6.57E-05	0.389
Docetaxel_1007	0.074	0.015487131	0.393	0.000217157	0.103
Gefitinib_1010	0.194	2.00E-10	0.089	0.419787194	-0.009
Navitoclax_1011	0.192	2.86E-10	0.419	7.35E-05	0.394
Vorinostat_1012	0.137	7.44E-06	0.406	0.000128901	0.154
Nilotinib_1013	0.253	6.78E-17	0.436	3.38E-05	0.430
Olaparib_1017	0.175	1.09E-08	0.480	3.85E-06	0.365
Axitinib_1021	0.215	1.42E-12	0.360	0.000757167	0.374
AZD7762_1022	0.081	0.008355489	0.453	1.49E-05	0.412
SB216763_1025	0.230	3.11E-14	0.444	2.31E-05	0.611
KU.55933_1030	0.136	9.00E-06	0.593	2.72E-09	0.409
Afatinib_1032	0.325	1.59E-27	0.317	0.003262083	0.064
Staurosporine_1034	0.071	0.021452329	0.454	1.47E-05	0.260
PLX.4720_1036	0.187	7.90E-10	0.467	7.71E-06	0.112
NU7441_1038	0.063	0.040993642	0.381	0.000352244	0.367
Doramapimod_1042	0.177	6.87E-09	0.162	0.140185703	0.552
Wee1.Inhibitor_1046	0.234	1.39E-14	0.430	4.36E-05	0.390
Nutlin.3a...._1047	0.104	0.000689402	0.323	0.002687147	0.223
Mirin_1048	0.177	6.84E-09	0.448	1.96E-05	0.155
PD173074_1049	0.276	6.49E-20	0.254	0.019897507	0.415
ZM447439_1050	0.183	2.05E-09	0.468	7.21E-06	0.379
Alisertib_1051	0.142	3.57E-06	0.409	0.00011004	0.254
RO.3306_1052	0.142	3.55E-06	0.301	0.00541383	0.389
MK.2206_1053	0.243	1.14E-15	0.375	0.000441815	0.261
Palbociclib_1054	0.186	1.10E-09	0.374	0.000458845	0.243
Dactolisib_1057	0.117	0.00013814	0.378	0.00039047	0.252
Pictilisib_1058	0.156	3.35E-07	0.374	0.000459493	0.091
AZD8055_1059	0.192	2.93E-10	0.675	1.93E-12	0.582
PD0325901_1060	0.068	0.028008072	0.199	0.069375647	-0.065
Obatoclax.Mesylate_1068	0.008	0.795552328	0.484	3.06E-06	0.282
X5.Fluorouracil_1073	0.155	3.76E-07	0.300	0.005562883	0.258
Dasatinib_1079	0.091	0.00317408	0.307	0.004444376	0.250
Paclitaxel_1080	0.167	4.63E-08	0.259	0.017294453	0.232
Crizotinib_1083	0.243	9.49E-16	0.321	0.002900477	0.438
Rapamycin_1084	0.190	4.15E-10	0.330	0.002197971	0.313
Sorafenib_1085	0.290	5.72E-22	0.218	0.045925436	0.316
BI.2536_1086	-0.084	0.006174623	-0.121	0.271022337	-0.028
Irinotecan_1088	0.143	2.87E-06	0.324	0.002672198	0.302
Oxaliplatin_1089	0.187	7.94E-10	0.466	8.10E-06	0.261
BMS.536924_1091	0.035	0.253994167	0.239	0.028739048	0.064
GSK1904529A_1093	0.292	2.72E-22	0.124	0.259987135	0.236
Tozasertib_1096	0.185	1.41E-09	0.305	0.004822879	0.285
PF.4708671_1129	0.208	7.98E-12	0.312	0.003909736	0.350
PRIMA.1MET_1131	0.231	2.57E-14	0.500	1.26E-06	0.571
Erlotinib_1168	0.131	1.97E-05	-0.028	0.797607416	-0.013
Niraparib_1177	0.256	2.32E-17	0.319	0.003149045	0.473
MK.1775_1179	0.199	6.17E-11	0.418	7.49E-05	0.393
Dinaciclib_1180	0.176	8.28E-09	0.177	0.106824555	0.250
Gemcitabine_1190	0.103	0.000831203	0.385	0.000300073	0.248

Bortezomib_1191	0.201	4.22E-11	0.541	1.07E-07	0.341
GSK269962A_1192	0.160	1.73E-07	0.423	5.98E-05	0.397
SB505124_1194	-0.179	4.70E-09	-0.338	0.001679309	-0.197
Tamoxifen_1199	0.047	0.122693939	0.244	0.025076636	-0.026
Fulvestrant_1200	0.319	2.10E-26	0.274	0.011683952	0.425
EPZ004777_1237	0.258	1.60E-17	0.391	0.00023782	0.504
YK.4.279_1239	0.197	9.69E-11	0.445	2.18E-05	0.298
Daporinad_1248	0.200	4.92E-11	0.332	0.002046505	0.283
BMS.345541_1249	0.204	2.21E-11	0.367	0.000598531	0.130
AZ960_1250	0.199	6.13E-11	0.470	6.53E-06	0.468
Talazoparib_1259	0.156	3.22E-07	0.476	4.63E-06	0.467
XAV939_1268	0.251	1.00E-16	0.114	0.299916186	0.367
Trametinib_1372	-0.003	0.933135313	0.213	0.051954786	-0.220
Dabrafenib_1373	0.176	7.37E-09	0.341	0.001505131	0.183
Temozolomide_1375	0.158	2.20E-07	0.418	7.67E-05	0.358
AZD5438_1401	0.155	4.18E-07	0.325	0.00255879	0.382
IAP_5620_1428	0.286	1.98E-21	0.216	0.047960664	0.444
AZD2014_1441	0.032	0.304578291	0.328	0.002354758	0.026
AZD1208_1449	0.250	1.69E-16	0.182	0.09675343	0.423
AZD1332_1463	0.038	0.218315297	0.313	0.003734022	0.152
Ruxolitinib_1507	0.388	1.91E-39	0.386	0.000287579	0.457
Linsitinib_1510	0.105	0.000647028	0.083	0.450778431	0.166
Epirubicin_1511	0.104	0.000676921	0.432	4.10E-05	0.279
Cyclophosphamide_1512	0.265	1.89E-18	0.453	1.50E-05	0.450
Pevonedistat_1529	0.126	3.92E-05	0.459	1.11E-05	0.338
Sapitinib_1549	0.172	1.68E-08	0.073	0.506600194	-0.145
Uprosertib_1553	0.187	9.32E-10	0.423	6.15E-05	0.062
LCL161_1557	0.318	2.33E-26	0.284	0.008943934	0.389
Lapatinib_1558	0.154	5.08E-07	0.246	0.024365051	-0.127
Luminespib_1559	0.184	1.66E-09	0.465	8.40E-06	0.253
Alpelisib_1560	0.293	2.19E-22	0.306	0.004633366	0.235
Taselisib_1561	0.217	9.92E-13	0.370	0.000529736	0.120
EPZ5676_1563	0.323	3.84E-27	0.365	0.000637439	0.454
SCH772984_1564	0.004	0.885670271	0.251	0.021537414	-0.230
IWP.2_1576	0.326	1.02E-27	0.504	1.03E-06	0.447
Leflunomide_1578	0.312	2.64E-25	0.190	0.083893914	0.545
Entinostat_1593	0.267	8.63E-19	0.363	0.000688848	0.356
OSI.027_1594	0.116	0.000157718	0.329	0.002266742	-0.078
LGK974_1598	0.322	6.01E-27	0.256	0.018916724	0.251
VE.822_1613	0.245	6.44E-16	0.524	3.12E-07	0.419
WZ4003_1614	0.078	0.011116658	0.417	7.93E-05	0.109
CZC24832_1615	0.255	3.88E-17	0.505	9.71E-07	0.463
AZD5582_1617	0.152	6.96E-07	0.280	0.009843931	0.420
GSK2606414_1618	0.290	6.29E-22	0.431	4.35E-05	0.350
PFI3_1620	0.303	5.44E-24	0.443	2.47E-05	0.481
PCI.34051_1621	0.330	2.26E-28	0.432	4.00E-05	0.552
Wnt.C59_1622	0.352	3.57E-32	0.273	0.011835341	0.361
I.BET.762_1624	0.110	0.000319246	0.374	0.000455422	0.349
RVX.208_1625	0.257	1.95E-17	0.450	1.75E-05	0.454
OTX015_1626	0.034	0.265202776	0.320	0.002967393	0.180
GSK343_1627	0.303	6.27E-24	0.533	1.77E-07	0.503
ML323_1629	0.338	1.02E-29	0.231	0.034592535	0.286
Entospletinib_1630	0.209	6.29E-12	0.507	8.59E-07	0.312
PRT062607_1631	0.180	4.00E-09	0.448	1.94E-05	0.332
Ribociclib_1632	0.262	4.96E-18	0.508	8.05E-07	0.534
AGI.6780_1634	0.314	1.16E-25	0.339	0.001593969	0.433

Picolinici.acid_1635	0.291	4.58E-22	0.513	6.24E-07	0.438
AZD5153_1706	0.006	0.832808445	0.120	0.275064019	0.103
CDK9_5576_1708	0.184	1.49E-09	0.208	0.057115225	0.317
CDK9_5038_1709	0.133	1.52E-05	0.155	0.158138432	0.251
Eg5_9814_1712	0.202	2.93E-11	0.294	0.006657594	0.162
ERK_2440_1713	-0.104	0.000740698	0.242	0.026872032	-0.295
ERK_6604_1714	-0.058	0.058676049	0.136	0.216490852	-0.299
IRAK4_4710_1716	0.189	5.28E-10	0.211	0.053461879	0.264
JAK1_8709_1718	0.143	3.12E-06	0.171	0.119739533	0.099
AZD5991_1720	0.296	8.68E-23	0.299	0.005662023	0.304
PAK_5339_1730	0.309	8.60E-25	0.492	2.05E-06	0.342
TAF1_5496_1732	0.290	5.63E-22	-0.174	0.113622234	0.147
ULK1_4989_1733	0.108	0.000444287	0.184	0.094538133	0.059
VSP34_8731_1734	0.153	5.38E-07	0.019	0.860939415	0.260
Selumetinib_1736	0.016	0.612982609	0.067	0.545637879	-0.028
IGF1R_3801_1738	0.058	0.061247361	0.135	0.221562127	0.203
JAK_8517_1739	0.173	1.45E-08	0.267	0.014157768	0.292
AZD4547_1786	0.213	2.61E-12	0.342	0.001466788	0.360
Ibrutinib_1799	0.321	8.70E-27	0.188	0.087330618	0.207
Zoledronate_1802	0.287	1.85E-21	0.363	0.000688217	0.430
Acetalax_1804	0.191	3.78E-10	0.061	0.580542748	-0.188
Oxaliplatin_1806	0.148	1.25E-06	0.292	0.006983196	0.219
Carmustine_1807	0.329	4.26E-28	0.403	0.000142091	0.352
Topotecan_1808	0.158	2.30E-07	0.335	0.001858134	0.356
Teniposide_1809	0.191	3.41E-10	0.389	0.000251711	0.445
Mitoxantrone_1810	0.188	7.14E-10	0.371	0.000517744	0.445
Dactinomycin_1811	0.225	1.29E-13	0.394	0.000212931	0.249
Fludarabine_1813	0.233	1.62E-14	0.522	3.55E-07	0.454
Nelarabine_1814	0.324	2.38E-27	0.348	0.001192376	0.460
Fulvestrant_1816	0.296	6.43E-23	0.030	0.783666872	0.280
Vincristine_1818	0.231	2.74E-14	0.425	5.68E-05	0.208
Docetaxel_1819	0.096	0.00183827	0.380	0.000360725	0.218
Podophyllotoxin.bromide_1825	0.200	4.97E-11	0.458	1.16E-05	0.322
Dihydrorotenone_1827	0.294	1.72E-22	-0.301	0.005475347	-0.008
Gallibiscoquinazole_1830	0.367	4.27E-35	0.206	0.060749243	0.425
Elephantin_1835	0.139	5.54E-06	0.309	0.004193611	0.162
Sinularin_1838	0.341	2.81E-30	0.294	0.0067346	0.197
Sabutoclax_1849	0.297	6.18E-23	0.388	0.00026498	0.390
LY2109761_1852	0.277	3.60E-20	0.367	0.00059417	0.366
OF.1_1853	0.383	2.51E-38	-0.102	0.357585531	0.262
MN.64_1854	0.363	2.42E-34	0.076	0.490180058	0.384
KRAS..G12C..Inhibitor.12_1855	0.362	3.96E-34	0.309	0.004188695	0.324
MG.132_1862	0.091	0.002939606	0.527	2.59E-07	0.206
BDP.00009066_1866	0.198	8.81E-11	0.449	1.87E-05	0.351
Buparlisib_1873	0.163	8.91E-08	0.422	6.46E-05	0.257
Ulixertinib_1908	0.036	0.242869394	0.293	0.006752076	-0.169
Venetoclax_1909	0.126	3.77E-05	0.467	7.46E-06	0.443
ABT737_1910	0.118	0.000120649	0.375	0.000436253	0.259
Dactinomycin_1911	-0.007	0.828162824	0.429	4.63E-05	-0.011
Afuresertib_1912	0.203	2.35E-11	0.447	2.03E-05	0.185
AGI.5198_1913	0.208	7.57E-12	0.501	1.21E-06	0.265
AZD3759_1915	0.217	9.64E-13	0.107	0.332332813	0.142
AZD5363_1916	0.205	1.65E-11	0.318	0.003242652	0.255
AZD6738_1917	0.173	1.48E-08	0.460	1.07E-05	0.383
AZD8186_1918	0.182	2.67E-09	0.391	0.000235257	0.274
Osimertinib_1919	0.294	1.70E-22	0.098	0.375341183	0.133

Cediranib_1922	0.162	1.06E-07	0.139	0.20596676	0.224
Ipatasertib_1924	0.215	1.40E-12	0.259	0.017280185	0.211
GDC0810_1925	0.282	9.65E-21	0.289	0.007615323	0.352
GNE.317_1926	0.138	6.51E-06	0.368	0.000575623	0.216
GSK2578215A_1927	0.224	1.57E-13	0.486	2.83E-06	0.424
I.BRD9_1928	0.223	2.08E-13	0.327	0.002367326	0.318
Telomerase.Inhibitor.IX_1930	0.215	1.41E-12	0.564	2.36E-08	0.272
MIRA.1_1931	0.311	3.69E-25	0.441	2.65E-05	0.424
NVP.ADW742_1932	0.044	0.152988647	0.391	0.00023102	0.009
P22077_1933	0.282	7.93E-21	0.394	0.000205759	0.346
Savolitinib_1936	0.232	1.87E-14	0.495	1.69E-06	0.448
UMI.77_1939	0.163	8.87E-08	0.375	0.000434347	0.248
WIKI4_1940	0.104	0.000717052	0.457	1.23E-05	0.181
Sepantronium.bromide_1941	-0.067	0.029852424	0.109	0.32298251	0.017
MIM1_1996	0.249	2.07E-16	0.544	9.12E-08	0.434
WEHI.539_1997	0.167	4.83E-08	0.468	7.17E-06	0.445
BPD.00008900_1998	0.230	3.48E-14	0.355	0.000906756	0.443
Foretinib_2040	0.036	0.235799052	0.330	0.002189942	0.236
BIBR.1532_2043	0.261	5.47E-18	0.478	4.17E-06	0.471
Pyridostatin_2044	0.214	2.19E-12	0.417	7.88E-05	0.354
AMG.319_2045	0.288	1.06E-21	0.469	6.72E-06	0.577
MK.8776_2046	0.149	1.03E-06	0.495	1.68E-06	0.482
Ulixertinib_2047	0.090	0.003322816	-0.036	0.743159097	0.094
Vinorelbine_2048	0.158	2.51E-07	0.454	1.41E-05	0.279
VX.11e_2096	0.043	0.16195592	0.298	0.005858061	-0.109
Uprosertib_2106	0.202	2.95E-11	0.284	0.008877902	0.175
LJI308_2107	0.218	6.87E-13	0.385	0.000302924	0.513
AZ6102_2109	0.179	4.74E-09	0.455	1.40E-05	0.342
GSK591_2110	0.291	3.79E-22	0.404	0.000138293	0.558
VE821_2111	0.263	3.59E-18	0.456	1.29E-05	0.338
AZD6482_2169	0.240	2.26E-15	0.368	0.000566518	0.539
AT13148_2170	0.237	5.56E-15	0.383	0.000324564	0.352
BMS.754807_2171	0.130	2.11E-05	0.444	2.28E-05	0.434
JQ1_2172	0.253	5.96E-17	0.367	0.000591533	0.552

SKCM	GBM		KIRC		LGG		
FDR	R	FDR	R	FDR	R	FDR	R
3.65E-12	-0.238	0.001977751	-0.233	5.78E-08	-0.159	0.000266801	0.296
4.90E-09	-0.005	0.952775329	-0.104	0.017230974	0.182	2.74E-05	0.181
2.62E-11	-0.088	0.258188192	-0.286	2.13E-11	-0.113	0.009721259	0.122
3.71E-16	-0.029	0.707256904	-0.087	0.045076291	0.018	0.686504108	0.182
0.03744661	0.033	0.668247748	-0.156	0.000321765	0.172	7.28E-05	-0.041
0.854319447	0.162	0.03646583	0.196	5.75E-06	0.343	6.93E-16	-0.192
1.63E-16	0.259	0.000739239	0.047	0.278610028	0.129	0.003046166	0.333
0.001885643	0.401	7.84E-08	-0.100	0.020973755	0.508	9.89E-36	0.442
1.17E-19	-0.032	0.685527776	-0.019	0.669793192	-0.017	0.695526513	0.274
2.82E-14	0.185	0.016928662	-0.049	0.261680021	0.208	1.50E-06	0.220
5.88E-15	-0.052	0.503514859	-0.164	0.000149588	-0.102	0.019544266	0.189
4.42E-18	-0.028	0.716437695	-0.206	1.78E-06	-0.184	2.32E-05	-0.205
7.28E-43	-0.200	0.009580161	-0.078	0.073249428	-0.209	1.39E-06	0.043
8.20E-18	-0.282	0.000218978	-0.231	7.60E-08	-0.460	9.33E-29	-0.264
0.1984984	0.061	0.433346322	0.186	1.77E-05	0.184	2.33E-05	-0.377
1.06E-07	-0.217	0.004823494	-0.076	0.082260999	-0.178	4.24E-05	-0.329
0.024382381	-0.292	0.000125246	-0.177	4.46E-05	-0.375	5.75E-19	0.100
2.07E-14	-0.100	0.199541143	0.031	0.482402659	-0.111	0.01092207	0.104
9.26E-34	0.215	0.005302325	0.180	3.19E-05	0.278	9.27E-11	0.424
3.49E-16	0.099	0.203106525	-0.023	0.599843992	0.203	2.74E-06	0.226
5.47E-06	-0.174	0.024843543	-0.040	0.354325692	0.004	0.934661486	0.264
0.001702986	-0.155	0.045326463	-0.273	1.85E-10	-0.185	2.04E-05	0.067
2.51E-18	-0.051	0.514323701	0.017	0.69830752	-0.115	0.008284306	0.179
2.68E-15	-0.091	0.241296429	-0.110	0.011161867	-0.285	3.22E-11	-0.177
2.13E-07	0.135	0.082607282	0.001	0.987922964	0.128	0.003328332	0.212
3.80E-16	0.013	0.869704384	0.014	0.750319507	-0.013	0.774632828	0.217
9.63E-08	-0.037	0.637314713	-0.026	0.556171026	0.040	0.355879205	0.258
7.02E-07	-0.015	0.847340675	-0.185	1.88E-05	0.143	0.001003658	0.244
2.69E-07	-0.132	0.089405882	-0.136	0.001679296	0.004	0.925362903	0.202
0.066681625	-0.111	0.151597177	-0.087	0.044861386	-0.060	0.17004289	-0.068
3.38E-38	-0.210	0.00653425	-0.351	1.00E-16	-0.405	4.11E-22	0.303
0.192441875	-0.285	0.000193488	0.140	0.001253093	-0.355	5.62E-17	-0.381
7.49E-09	0.051	0.512344555	-0.078	0.075009057	-0.008	0.852915085	0.122
1.42E-07	-0.306	5.71E-05	-0.189	1.22E-05	-0.304	1.21E-12	-0.175
3.39E-07	-0.355	2.43E-06	-0.072	0.097466372	-0.308	5.88E-13	-0.367
2.20E-06	-0.057	0.462269095	-0.045	0.302820783	0.171	8.05E-05	0.002
1.97E-20	-0.140	0.072124781	-0.032	0.459621251	-0.147	0.000735145	-0.021
1.07E-10	-0.250	0.001136418	-0.151	0.000507498	-0.223	2.62E-07	0.198
7.48E-11	0.040	0.604135812	-0.129	0.00301931	0.060	0.17375884	0.417
0.575073597	0.256	0.000854019	0.261	1.07E-09	0.307	7.04E-13	0.072
5.45E-10	-0.112	0.150684346	-0.176	4.74E-05	-0.070	0.107668307	0.334
1.00E-07	-0.078	0.316966414	-0.055	0.205691494	0.043	0.320621381	0.318
0.200266563	-0.089	0.250487778	-0.043	0.321612071	0.035	0.425248665	0.142
1.56E-06	0.084	0.28081935	-0.011	0.795673984	0.132	0.002549995	0.310
5.29E-09	0.003	0.971668976	-0.043	0.322683894	-0.037	0.399773564	0.107
3.75E-13	-0.115	0.138370557	-0.286	2.00E-11	-0.368	2.80E-18	0.069
1.83E-36	0.112	0.148820533	-0.231	8.17E-08	0.059	0.178379758	0.297
0.786870431	0.197	0.010902262	0.291	9.05E-12	0.412	6.16E-23	-0.231
4.91E-24	0.029	0.706474085	-0.049	0.263689759	0.099	0.023555174	0.244
1.76E-16	0.013	0.866137126	-0.062	0.156268	0.030	0.488216857	-0.010
3.27E-07	-0.202	0.008801559	-0.059	0.175458318	-0.193	8.39E-06	-0.279
4.40E-07	-0.204	0.008160399	-0.205	2.11E-06	-0.210	1.31E-06	0.120

1.68E-12	-0.194	0.012118433	-0.101	0.020181783	-0.209	1.46E-06	-0.129
8.47E-17	-0.039	0.616710127	-0.210	1.14E-06	-0.278	9.70E-11	0.254
6.45E-05	0.200	0.009513798	0.476	3.82E-31	0.512	2.32E-36	0.104
0.600739309	0.324	1.96E-05	-0.090	0.039443209	0.382	1.07E-19	0.312
3.08E-19	-0.036	0.641838224	-0.144	0.000904191	0.066	0.131136013	0.252
1.50E-27	0.022	0.781801743	-0.161	0.000211172	-0.155	0.000384637	0.239
8.99E-10	-0.038	0.626070433	0.015	0.724175662	0.132	0.002536861	0.002
6.40E-09	0.160	0.039301962	0.076	0.079443073	0.310	3.85E-13	0.088
0.008776937	-0.159	0.039763702	0.049	0.262156885	0.028	0.524545903	0.034
1.90E-23	-0.221	0.004131302	-0.195	6.58E-06	-0.165	0.000144129	0.030
2.12E-23	-0.101	0.192398817	-0.046	0.288151339	-0.012	0.784499201	0.227
2.24E-14	-0.252	0.001015898	-0.354	4.91E-17	-0.421	5.55E-24	-0.031
7.43E-06	-0.298	9.33E-05	0.158	0.000266343	-0.235	5.26E-08	-0.459
0.000204166	-0.007	0.925014726	-0.311	2.76E-13	-0.085	0.051736958	0.256
1.04E-13	-0.017	0.822397026	-0.149	0.000572563	0.044	0.316572076	0.256
1.48E-15	-0.239	0.001898666	-0.101	0.019679896	-0.270	3.53E-10	-0.247
5.08E-21	-0.264	0.000553078	0.253	3.83E-09	-0.374	8.53E-19	0.257
0.596523906	-0.324	1.92E-05	-0.211	9.96E-07	-0.240	2.66E-08	0.074
4.34E-19	0.196	0.01100517	0.106	0.015170203	0.129	0.003078643	0.356
0.002167927	-0.254	0.000920633	-0.136	0.001693275	-0.419	1.09E-23	0.074
2.65E-22	-0.162	0.036489036	-0.029	0.507032559	-0.147	0.000729501	0.138
0.000799269	0.190	0.013771368	-0.053	0.222865377	0.473	1.57E-30	0.317
1.07E-08	-0.089	0.252371243	-0.224	1.90E-07	0.036	0.414956577	0.307
1.14E-21	0.127	0.101237766	-0.112	0.009924382	0.167	0.000126449	0.288
2.74E-12	-0.184	0.017241662	-0.026	0.552444782	-0.013	0.764484613	0.173
0.003323213	0.019	0.808008602	0.166	0.000128863	-0.034	0.437327723	-0.480
0.211959852	-0.129	0.097045218	-0.032	0.46241354	-0.141	0.00124989	0.160
3.86E-16	-0.138	0.074646802	0.234	5.45E-08	-0.145	0.000879499	0.250
0.010505819	0.206	0.007647494	-0.174	6.12E-05	0.421	6.18E-24	-0.323
2.28E-07	-0.262	0.000641114	-0.151	0.000493451	-0.153	0.000434709	-0.054
1.66E-06	-0.153	0.048959778	-0.105	0.016113263	-0.229	1.15E-07	-0.186
0.015776021	-0.127	0.100809112	-0.099	0.022675535	-0.181	2.92E-05	-0.239
5.43E-22	-0.035	0.652408875	0.039	0.377127962	-0.134	0.002144648	0.250
2.96E-06	-0.365	1.24E-06	0.139	0.001311113	-0.321	5.08E-14	-0.499
2.37E-21	-0.016	0.841715455	-0.094	0.031374989	-0.106	0.015464163	0.237
8.88E-33	0.057	0.461393668	-0.284	2.99E-11	0.119	0.00650331	0.371
1.32E-13	0.223	0.003773037	-0.115	0.008110801	0.280	7.12E-11	0.228
0.114521607	0.063	0.415542029	-0.097	0.025211357	-0.025	0.564591537	0.027
2.96E-07	-0.077	0.322529088	-0.138	0.001504441	-0.039	0.37419904	0.233
1.15E-18	0.103	0.186925847	-0.237	3.36E-08	-0.140	0.001279014	-0.209
0.028748364	-0.030	0.698639703	-0.190	1.08E-05	-0.140	0.001353942	0.084
5.20E-23	-0.268	0.00045732	-0.097	0.025960222	-0.382	1.34E-19	0.108
8.07E-19	-0.272	0.000377979	0.009	0.843785097	-0.311	3.26E-13	-0.207
3.92E-13	-0.232	0.002559698	0.052	0.232244534	-0.277	1.09E-10	0.156
6.49E-25	-0.075	0.33264774	0.004	0.923630581	-0.174	6.30E-05	0.155
8.09E-34	0.058	0.453687509	0.187	1.54E-05	0.156	0.00032813	0.299
5.78E-14	0.092	0.234748617	-0.040	0.36411802	0.126	0.003960867	0.353
4.54E-13	-0.295	0.000108509	-0.052	0.232233503	-0.101	0.020140834	0.145
4.65E-22	-0.226	0.003249153	-0.052	0.236016012	-0.205	2.32E-06	0.127
0.000272182	-0.227	0.003112404	-0.171	7.57E-05	-0.125	0.004310004	-0.021
2.02E-27	-0.156	0.044370399	-0.068	0.117114107	-0.181	3.13E-05	0.117
4.40E-09	-0.016	0.836518142	0.061	0.163964121	0.109	0.012150564	0.275
1.30E-10	-0.413	2.82E-08	-0.185	1.81E-05	-0.444	9.23E-27	-0.123
7.05E-12	-0.061	0.436744532	-0.204	2.39E-06	-0.008	0.85930174	0.090
2.66E-31	-0.186	0.015964394	-0.299	2.33E-12	-0.328	1.44E-14	0.091
5.20E-20	0.019	0.80797091	0.011	0.806861365	-0.024	0.577308826	0.268

1.77E-20	-0.149	0.053842497	-0.064	0.140048998	-0.240	2.82E-08	0.219
0.037104417	-0.105	0.177352256	-0.144	0.000897706	0.078	0.073706206	0.213
6.38E-11	-0.225	0.003397742	-0.074	0.089095089	-0.156	0.000338941	-0.188
2.85E-07	-0.177	0.02236838	-0.241	1.93E-08	-0.130	0.002839414	-0.124
0.001080198	0.081	0.295600431	0.059	0.178063913	0.234	5.65E-08	0.179
1.27E-09	-0.403	6.69E-08	-0.236	3.81E-08	-0.341	1.07E-15	-0.317
8.05E-10	-0.289	0.000151835	-0.232	7.04E-08	-0.244	1.45E-08	-0.484
6.43E-08	-0.010	0.900587125	-0.146	0.000780396	-0.184	2.36E-05	0.020
0.046197666	-0.157	0.042690494	-0.011	0.80412186	-0.116	0.007993438	0.329
3.83E-10	0.039	0.620333937	0.091	0.036737862	0.074	0.091871826	0.149
1.42E-12	-0.035	0.648840991	-0.078	0.073792838	-0.032	0.469420542	0.107
0.003042725	0.280	0.000250505	0.099	0.023382459	0.178	4.22E-05	0.402
0.234071772	-0.333	1.08E-05	-0.324	2.37E-14	-0.280	6.33E-11	-0.357
1.04E-07	-0.317	2.97E-05	-0.232	6.53E-08	-0.354	6.98E-17	-0.394
0.574030397	-0.294	0.000114459	0.033	0.451985163	-0.377	3.69E-19	-0.452
3.74E-05	0.015	0.84315807	-0.095	0.029111959	0.029	0.503276113	-0.021
2.03E-09	-0.120	0.123285119	-0.085	0.05051018	-0.256	2.89E-09	0.124
7.16E-14	-0.042	0.589819599	0.119	0.006041242	-0.046	0.29362624	0.348
2.55E-05	0.010	0.897872376	0.151	0.000510306	-0.061	0.163620696	-0.280
9.79E-20	0.039	0.619047625	0.136	0.001759031	-0.046	0.297798071	0.332
0.000133555	-0.088	0.25623668	0.064	0.144625366	-0.112	0.01047741	-0.492
8.83E-06	-0.048	0.535066916	-0.013	0.758328442	-0.012	0.775999031	0.287
2.84E-13	0.077	0.324537405	0.088	0.04365534	0.021	0.630669934	0.218
1.43E-13	-0.196	0.011255126	-0.318	7.36E-14	-0.173	7.10E-05	0.236
3.60E-21	-0.122	0.117675192	-0.195	6.17E-06	-0.247	1.03E-08	0.283
4.02E-21	-0.125	0.107585908	-0.112	0.009787883	-0.091	0.037527777	0.298
3.92E-07	-0.299	8.79E-05	-0.063	0.150148975	-0.202	3.00E-06	0.164
4.84E-22	-0.036	0.641137073	-0.040	0.364256223	-0.071	0.102918567	0.084
1.12E-22	0.082	0.290016214	0.074	0.088840258	-0.033	0.447160609	0.181
9.76E-09	0.177	0.022210003	-0.086	0.04939197	0.128	0.003264471	0.319
2.30E-05	-0.045	0.559716786	-0.073	0.092527076	0.029	0.50783671	0.123
9.68E-06	-0.056	0.473469694	-0.039	0.370832685	0.022	0.60988624	-0.191
3.05E-11	0.114	0.141563925	-0.123	0.004617588	0.153	0.000439815	0.225
0.875846562	0.150	0.052447625	0.157	0.000304513	0.143	0.000998658	0.292
3.15E-19	0.052	0.506958758	0.089	0.040831732	0.057	0.193070771	0.298
0.00106904	0.187	0.01570499	-0.297	3.21E-12	0.236	4.64E-08	0.361
6.60E-05	0.059	0.445385548	0.231	8.39E-08	0.123	0.004829414	0.045
3.21E-16	0.027	0.730468471	-0.232	7.13E-08	0.064	0.140965539	0.324
2.67E-14	0.061	0.436404102	0.186	1.65E-05	0.094	0.032031015	0.158
8.06E-08	0.118	0.129176449	0.286	2.14E-11	0.279	7.38E-11	0.314
9.48E-16	-0.033	0.673315067	0.007	0.872929865	-0.057	0.191725924	0.066
2.24E-11	-0.012	0.877537692	-0.029	0.51022771	0.001	0.987308928	0.134
2.80E-05	-0.144	0.063125446	-0.182	2.57E-05	-0.161	0.000209802	-0.096
3.13E-13	-0.284	0.000203512	-0.045	0.30585992	-0.100	0.021758549	-0.034
1.52E-07	-0.202	0.008919256	-0.019	0.657322394	-0.015	0.740357672	0.139
0.000638481	-0.193	0.012616527	0.159	0.000244735	0.060	0.171082645	-0.250
6.13E-21	0.240	0.001796162	0.126	0.003674908	0.222	2.73E-07	0.359
1.20E-07	0.393	1.45E-07	0.195	6.66E-06	0.224	2.23E-07	0.367
0.822953841	-0.027	0.733735768	-0.220	3.39E-07	0.146	0.000816905	0.136
0.000176625	-0.011	0.882794203	-0.110	0.011433668	0.019	0.66945704	0.198
5.72E-08	0.111	0.151753231	-0.395	3.35E-21	0.220	3.82E-07	0.316
0.004094379	0.162	0.036433547	0.221	3.02E-07	0.165	0.000148977	-0.280
1.92E-07	-0.156	0.044462396	-0.155	0.000355267	-0.173	7.12E-05	0.087
1.32E-15	-0.023	0.766267783	-0.214	6.58E-07	-0.084	0.054434806	-0.194
2.00E-08	-0.278	0.000274463	-0.058	0.184915632	-0.307	6.62E-13	-0.188
0.007087302	0.025	0.749580761	0.148	0.000671671	0.185	2.00E-05	-0.184

5.00E-06	-0.072	0.351881952	0.009	0.842127781	-0.196	5.88E-06	-0.073
1.73E-05	-0.091	0.24419068	-0.078	0.0738824	-0.079	0.069962551	0.093
2.88E-13	0.078	0.317937761	-0.115	0.008238061	0.194	7.75E-06	0.224
1.14E-05	-0.132	0.089830418	-0.161	0.000201234	-0.031	0.477320451	-0.039
3.82E-19	0.204	0.008037005	-0.265	6.52E-10	0.173	7.19E-05	0.326
5.24E-11	0.024	0.761481914	-0.102	0.018537149	0.253	4.20E-09	0.327
2.59E-08	-0.026	0.742070472	-0.165	0.000136892	-0.018	0.689320133	0.236
3.48E-19	0.085	0.277250032	-0.104	0.016974998	0.068	0.118911383	0.380
0.861374472	0.173	0.025578063	0.066	0.128675005	0.533	7.25E-40	0.371
7.23E-13	0.081	0.298780312	0.181	2.96E-05	0.257	2.26E-09	0.358
1.78E-21	-0.233	0.002431211	-0.154	0.000387856	-0.120	0.006159265	0.180
3.96E-07	0.217	0.004908659	0.203	2.51E-06	0.222	2.90E-07	0.111
0.000249013	-0.136	0.079809056	-0.281	5.13E-11	-0.193	8.38E-06	-0.246
0.736015128	0.007	0.933251985	0.003	0.93828435	0.047	0.286598968	0.069
4.64E-20	-0.041	0.601338427	-0.026	0.550190529	0.026	0.545654277	0.148
4.14E-21	0.350	3.43E-06	0.105	0.015912353	0.171	8.15E-05	0.071
5.51E-21	-0.253	0.000967356	-0.169	9.79E-05	-0.198	5.08E-06	-0.033
1.44E-06	0.007	0.92941334	-0.142	0.001067675	0.049	0.258758823	0.195
9.14E-24	0.254	0.000938099	0.054	0.218094676	0.324	3.05E-14	0.427
2.14E-13	0.158	0.041297969	-0.165	0.000141427	0.227	1.44E-07	0.328
2.41E-37	-0.207	0.007358347	-0.148	0.00066385	-0.281	5.82E-11	0.245
5.92E-25	-0.026	0.735848978	-0.261	1.11E-09	-0.153	0.000459607	0.097
0.058224927	-0.032	0.678095553	0.060	0.166487951	0.213	8.98E-07	-0.041
1.04E-08	-0.053	0.494968348	-0.127	0.00339285	0.048	0.26974104	0.067
0.028581984	-0.204	0.008086011	-0.051	0.244533936	-0.152	0.000493507	-0.383
0.000385599	-0.044	0.569933675	-0.118	0.006608233	-0.030	0.491206515	0.203
1.13E-28	0.038	0.630142559	-0.079	0.067987012	0.033	0.448000837	0.261
1.44E-12	0.009	0.911384716	0.209	1.29E-06	-0.011	0.795202309	0.140
1.51E-34	-0.043	0.580938922	-0.114	0.008888473	-0.028	0.528722655	0.197
2.83E-12	-0.064	0.412136743	-0.104	0.017348434	-0.142	0.001159647	-0.015
6.11E-32	-0.029	0.709872809	-0.205	2.16E-06	-0.293	8.06E-12	0.054
2.59E-13	-0.107	0.16967119	0.019	0.655219078	-0.052	0.235403428	0.264
4.13E-20	-0.079	0.312517642	-0.118	0.006739516	-0.005	0.91023943	0.217
9.14E-34	0.004	0.961719798	-0.089	0.041107193	0.016	0.716961736	0.114

PAAD	UVM	
FDR	R	FDR
7.17E-05	0.144	0.204882881
0.01692168	0.113	0.319292298
0.109071949	-0.048	0.675175881
0.016476722	0.188	0.097416833
0.589854222	0.293	0.008846682
0.01106026	0.245	0.029656781
7.14E-06	-0.002	0.988938938
9.96E-10	0.136	0.233517561
0.000253855	0.039	0.731694423
0.003490492	-0.097	0.397357514
0.012389807	0.012	0.916640783
0.006706764	-0.019	0.869160904
0.576033445	0.055	0.632950355
0.000433329	-0.155	0.172756003
3.00E-07	0.174	0.125842742
9.13E-06	0.097	0.395209147
0.18727255	-0.072	0.530366824
0.173847455	0.068	0.553792703
5.74E-09	0.063	0.583948807
0.00269138	0.115	0.314882719
0.000434143	-0.028	0.80633779
0.382742733	0.111	0.33214443
0.018216489	0.042	0.711328502
0.019203772	0.041	0.718379158
0.005021842	0.169	0.137564482
0.003978842	0.093	0.414243828
0.00058631	0.017	0.884485355
0.001161099	-0.080	0.484139855
0.007602189	0.076	0.505337636
0.372478361	0.062	0.584536079
4.77E-05	-0.083	0.466163969
2.09E-07	0.228	0.043574394
0.108207676	-0.026	0.819701912
0.02127764	-0.047	0.682196328
6.44E-07	0.003	0.981502777
0.976950252	0.116	0.30850395
0.781932553	0.102	0.370045053
0.008926972	-0.044	0.702865883
1.05E-08	0.060	0.598656579
0.343557517	0.231	0.040946756
6.72E-06	0.136	0.23354189
1.85E-05	0.027	0.813723109
0.062183238	0.029	0.802741856
3.15E-05	0.077	0.502633615
0.158465078	0.070	0.54144591
0.362321917	-0.220	0.051028937
7.06E-05	0.048	0.675847618
0.00219925	0.114	0.31595813
0.001152761	0.030	0.791041691
0.899830708	0.060	0.601781686
0.000191104	0.131	0.25041253
0.113827638	0.131	0.249488274

0.090109702	-0.037	0.748708841
0.000711967	-0.120	0.293945565
0.171448469	0.287	0.010359563
2.72E-05	-0.084	0.463356029
0.00078102	0.060	0.599189938
0.001504096	0.100	0.38054981
0.980232553	0.081	0.475775134
0.249190272	0.163	0.150932971
0.659577367	0.025	0.827508616
0.698221478	-0.095	0.405156508
0.00262717	0.067	0.55991934
0.689164669	-0.235	0.036884853
1.88E-10	0.249	0.027132238
0.000664535	-0.193	0.088696332
0.000663428	-0.032	0.781317899
0.001009303	-0.046	0.684105047
0.000615296	0.249	0.027187809
0.331007194	-0.126	0.268096064
1.45E-06	0.099	0.384889286
0.329825313	-0.098	0.389869201
0.069191026	0.026	0.818961047
2.09E-05	0.114	0.315181151
3.79E-05	0.292	0.009118185
0.0001164	0.041	0.717826945
0.022204025	0.052	0.647520173
2.02E-11	0.166	0.143692512
0.034532155	0.078	0.492821343
0.000899547	0.268	0.01704083
1.36E-05	0.031	0.785146417
0.477175	0.097	0.396236635
0.013825737	0.059	0.602867528
0.001493702	0.018	0.876858414
0.000883387	0.090	0.430256363
2.42E-12	0.267	0.017306626
0.001610923	0.094	0.408474843
4.82E-07	0.093	0.415583341
0.002505571	0.123	0.278911939
0.727117338	-0.020	0.859704467
0.00193963	-0.039	0.73406921
0.005691248	0.023	0.843672713
0.271064142	-0.095	0.402478593
0.156780157	0.042	0.712055632
0.006239487	0.145	0.203212069
0.040178537	0.039	0.734743348
0.041273601	0.072	0.526903898
6.07E-05	0.109	0.337886798
1.78E-06	0.149	0.189472444
0.055565833	0.156	0.170264572
0.093912468	-0.083	0.468660199
0.783377164	0.159	0.162505328
0.124092347	0.022	0.848359473
0.000242048	0.055	0.630593742
0.105191167	0.085	0.455711823
0.239255624	0.102	0.369694855
0.231426827	-0.113	0.319865562
0.000349772	0.050	0.66268868

0.00369365	0.007	0.953568893
0.004753048	0.201	0.075066868
0.012832912	0.035	0.759284819
0.103261906	0.077	0.49888402
0.018098042	0.193	0.089079249
2.00E-05	0.017	0.881909479
1.35E-11	0.150	0.186495855
0.797413681	0.043	0.706008275
9.24E-06	-0.027	0.812315379
0.04917263	0.052	0.646675274
0.158179467	0.024	0.836592656
3.76E-08	0.018	0.874722638
1.37E-06	0.124	0.276884701
7.49E-08	0.005	0.962779446
3.94E-10	0.101	0.37495499
0.785300472	0.125	0.272087317
0.102501282	0.006	0.961547781
2.47E-06	-0.141	0.21497033
0.000182595	0.105	0.356468595
7.61E-06	0.030	0.79544953
5.71E-12	-0.040	0.729251279
0.000124388	0.019	0.865308857
0.003889119	0.141	0.215591298
0.001717394	0.019	0.864727844
0.000158778	0.138	0.226836028
6.69E-05	0.151	0.182660498
0.030942678	0.233	0.038584355
0.268809579	0.088	0.442294827
0.016909745	0.164	0.148157563
1.81E-05	0.161	0.156310557
0.1071513	0.097	0.394500333
0.01150069	0.197	0.081929469
0.002872048	0.177	0.117939512
9.04E-05	0.191	0.090983231
6.64E-05	0.139	0.222997173
9.83E-07	0.063	0.579566892
0.558668623	0.286	0.010524013
1.31E-05	0.077	0.498291459
0.036964913	0.057	0.615113284
2.41E-05	0.104	0.361360823
0.384475672	0.037	0.74633626
0.077157243	0.077	0.50188797
0.206191024	0.054	0.6355085
0.660613742	0.027	0.810592365
0.067288413	0.036	0.754231567
0.00087071	0.172	0.129308152
1.18E-06	-0.081	0.478362684
6.38E-07	-0.140	0.217166261
0.074400509	0.267	0.017368742
0.008904561	0.104	0.36010681
2.21E-05	-0.084	0.460384819
0.000184823	0.196	0.084051922
0.256384139	0.063	0.582925751
0.010256794	-0.007	0.952321288
0.013222564	-0.077	0.498695789
0.015067291	0.076	0.505055013

0.336298223	0.105	0.358656586
0.223687793	0.027	0.812304191
0.002928995	0.143	0.207073846
0.604999022	-0.006	0.961473984
1.13E-05	0.090	0.42898603
1.03E-05	0.072	0.529529835
0.001729211	0.132	0.244832822
2.24E-07	0.151	0.184757468
4.59E-07	0.141	0.216761235
1.22E-06	0.044	0.700237035
0.017598989	0.042	0.709971216
0.145673069	0.120	0.293150065
0.001047787	-0.220	0.051030152
0.365590115	0.306	0.006068436
0.050904302	0.077	0.50172623
0.349231072	-0.056	0.626992249
0.662244025	0.024	0.832616579
0.009781209	0.066	0.560654217
4.22E-09	0.136	0.231767606
1.03E-05	0.163	0.151786814
0.001124644	0.019	0.864978392
0.201755283	0.005	0.965172569
0.593339078	0.250	0.026283865
0.381455155	0.076	0.507150757
1.84E-07	0.239	0.033807482
0.007210711	0.098	0.388303806
0.000511484	0.007	0.948125577
0.065390532	0.208	0.065275081
0.009050719	0.231	0.040472507
0.84092749	0.035	0.759669915
0.480825501	0.107	0.349232384
0.000431056	0.076	0.506598602
0.003997423	-0.048	0.672149561
0.135861155	0.034	0.763796038
